# Genetic copy number variants, cognition and psychosis: a meta-analysis and a family study

**DOI:** 10.1038/s41380-020-0820-7

**Published:** 2020-07-27

**Authors:** Johan H. Thygesen, Amelia Presman, Jasmine Harju-Seppänen, Haritz Irizar, Rebecca Jones, Karoline Kuchenbaecker, Kuang Lin, Behrooz Z. Alizadeh, Isabelle Austin-Zimmerman, Agna Bartels-Velthuis, Anjali Bhat, Richard Bruggeman, Wiepke Cahn, Stella Calafato, Benedicto Crespo-Facorro, Liewe de Haan, Sonja M. C. de Zwarte, Marta Di Forti, Álvaro Díez-Revuelta, Jeremy Hall, Mei-Hua Hall, Conrad Iyegbe, Assen Jablensky, Rene Kahn, Luba Kalaydjieva, Eugenia Kravariti, Stephen Lawrie, Jurjen J. Luykx, Igancio Mata, Colm McDonald, Andrew M. McIntosh, Andrew McQuillin, Rebecca Muir, Roel Ophoff, Marco Picchioni, Diana P. Prata, Siri Ranlund, Dan Rujescu, Bart P. F. Rutten, Katja Schulze, Madiha Shaikh, Frederike Schirmbeck, Claudia J. P. Simons, Timothea Toulopoulou, Therese van Amelsvoort, Neeltje van Haren, Jim van Os, Ruud van Winkel, Evangelos Vassos, Muriel Walshe, Matthias Weisbrod, Eirini Zartaloudi, Vaughan Bell, John Powell, Cathryn M. Lewis, Robin M. Murray, Elvira Bramon

**Affiliations:** 1 Division of Psychiatry, University College London, London, UK; 2 UCL Genetics Institute, University College London, London, UK; 3 Institute of Psychiatry, Psychology & Neuroscience at King’s College London, London, UK; 4 Nuffield Department of Population Health, University of Oxford, Oxford, UK; 5 University of Groningen, University Medical Center Groningen, University Center for Psychiatry, Rob Giel Research Center, Groningen, The Netherlands; 6 Department of Epidemiology, University Medical Center Groningen, Groningen, The Netherlands; 7 Department of Clinical and Developmental Neuropsychology, University of Groningen, Groningen, The Netherlands; 8 University Medical Center Utrecht, Department of Psychiatry, Brain Centre Rudolf Magnus, Utrecht University, Utrecht, The Netherlands; 9 Altrecht, General Mental Health Care, Utrecht, The Netherlands; 10 CIBERSAM, Centro Investigación Biomédica en Red Salud Mental, Sevilla, Spain; 11 University Hospital Marqués de Valdecilla, University of Cantabria–IDIVAL, Santander, Spain; 12 Hospital Universitario Virgen del Rocío, IBiS, Department of Psychiatry, School of Medicine, University of Sevilla, Sevilla, Spain; 13 Amsterdam UMC, Department of Psychiatry, University of Amsterdam, Meibergdreef 9, Amsterdam, The Netherlands; 14 Arkin, Institute for Mental Health, Amsterdam, The Netherlands; 15 Laboratory of Cognitive and Computational Neuroscience—Centre for Biomedical Technology (CTB), Complutense University and Technical University of Madrid, Madrid, Spain; 16 School of Medicine, Cardiff University, Hadyn Ellis Building, Maindy Road, Cardiff, UK; 17 Psychosis Neurobiology Laboratory, Harvard Medical School, McLean Hospital, Belmont, MA USA; 18 Centre for Clinical Research in Neuropsychiatry, The University of Western Australia, Perth, WA Australia; 19 Department of Psychiatry, Icahn School of Medicine at Mount Sinai, New York, NY USA; 20 Harry Perkins Institute of Medical Research and Centre for Medical Research, The University of Western Australia, Perth, WA Australia; 21 Division of Psychiatry, University of Edinburgh, Royal Edinburgh Hospital, Edinburgh, Scotland UK; 22 Department of Translational Neuroscience, Brain Center Rudolf Magnus, University Medical Center Utrecht, Utrecht, The Netherlands; 23 Second opinion outpatient clinic, GGNet Mental Health, Warsnveld, The Netherlands; 24 Fundación Argibide, Pamplona, Spain; 25 The Centre for Neuroimaging & Cognitive Genomics (NICOG) and NCBES Galway Neuroscience Centre, National University of Ireland Galway, Galway, Ireland; 26 Centre for Cognitive Ageing and Cognitive Epidemiology, University of Edinburgh, Edinburgh, UK; 27 Center for Neurobehavioral Genetics, Semel Institute for Neuroscience and Human Behavior, University of California Los Angeles, Los Angeles, CA USA; 28 Department of Human Genetics, David Geffen School of Medicine, University of California Los Angeles, Los Angeles, CA USA; 29 Department of Psychiatry, Erasmus MC University Medical Center Rotterdam, Rotterdam, The Netherlands; 30 Instituto de Biofísica e Engenharia Biomédica, Faculdade de Ciencias da Universidade de Lisboa, Lisboa, Portugal; 31 Centre for Psychological Research and Social Intervention, ISCTE-IUL, Lisboa, Portugal; 32 Department of Psychiatry, Ludwig-Maximilians University of Munich, Munich, Germany; 33 Department of Psychiatry, Psychotherapy and Psychosomatics, University of Halle Wittenberg, Halle, Germany; 34 Department of Psychiatry & Neuropsychology, School for Mental Health and Neuroscience, Maastricht University Medical Centre, Maastricht, The Netherlands; 35 The Brain+Nerve Centre, Maastricht University Medical Centre+ (MUMC+), Maastricht, The Netherlands; 36 South London and Maudsley NHS Foundation Trust, London, UK; 37 North East London Foundation Trust, London, UK; 38 Research Department of Clinical, Educational and Health Psychology, University College London, London, UK; 39 GGzE Institute for Mental Health Care, Eindhoven, The Netherlands; 40 Department of Psychology, Bilkent University, Main Campus, Bilkent, Ankara Turkey; 41 Department of Child and Adolescent Psychiatry/Psychology, Erasmus University Medical Center, Sophia’s Children Hospital, Rotterdam, The Netherlands; 42 Department of Psychiatry, UMC Utrecht Brain Center, Utrecht, The Netherlands; 43 KU Leuven, Department of Neuroscience, Research Group Psychiatry, Leuven, Belgium; 44 Department of General Psychiatry, Center of Psychosocial Medicine, University of Heidelberg, Heidelberg, Germany; 45 SRH Klinikum, Karlsbad-Langensteinbach, Germany; 46 Institute of Cognitive Neuroscience, University College London, London, UK

**Keywords:** Predictive markers, Genetics, Neuroscience

## Abstract

The burden of large and rare copy number genetic variants (CNVs) as well as certain specific CNVs increase the risk of developing schizophrenia. Several cognitive measures are purported schizophrenia endophenotypes and may represent an intermediate point between genetics and the illness. This paper investigates the influence of CNVs on cognition. We conducted a systematic review and meta-analysis of the literature exploring the effect of CNV burden on general intelligence. We included ten primary studies with a total of 18,847 participants and found no evidence of association. In a new psychosis family study, we investigated the effects of CNVs on specific cognitive abilities. We examined the burden of large and rare CNVs (>200 kb, <1% MAF) as well as known schizophrenia-associated CNVs in patients with psychotic disorders, their unaffected relatives and controls (*N* = 3428) from the Psychosis Endophenotypes International Consortium (PEIC). The carriers of specific schizophrenia-associated CNVs showed poorer performance than non-carriers in immediate (*P* = 0.0036) and delayed (*P* = 0.0115) verbal recall. We found suggestive evidence that carriers of schizophrenia-associated CNVs had poorer block design performance (*P* = 0.0307). We do not find any association between CNV burden and cognition. Our findings show that the known high-risk CNVs are not only associated with schizophrenia and other neurodevelopmental disorders, but are also a contributing factor to impairment in cognitive domains such as memory and perceptual reasoning, and act as intermediate biomarkers of disease risk.

## Introduction

Copy number variants (CNVs) occur if sections of DNA sequence become deleted or duplicated [1–3]. Although many CNVs are benign and contribute to natural human variation [4], larger and rarer variants are more likely to be pathogenic and under negative selection pressure [5, 6]. The phenotypic effects of CNVs are not fully understood, but they influence neurodevelopment, cognitive abilities and the risk of several common brain disorders [6].

Specific CNV loci are associated with increased risk of developing schizophrenia [7–11]. A recent large CNV meta-analysis by the Psychiatric Genomics Consortium showed robust genome-wide significant associations for eight loci as well as suggestive support for an additional nine [12]. Schizophrenia-associated CNVs have incomplete penetrance [6, 13], and are rare, hence most people with schizophrenia are not carriers. However, schizophrenia-associated CNVs have odds ratios ranging from 2 to 30 [12, 14] and thus constitute some of the strongest known risk factors for the illness.

An increased burden of large and rare CNVs has also been associated with schizophrenia [15, 16]. Studies have shown that, compared with healthy controls, individuals with schizophrenia carry a greater number of rare (<1% frequency) CNVs of over 20 kilobases (kb) [12], 100 kb [15–17], 200 kb [16, 17], 500 kb [16, 17] and 1 Mb [18]. The largest study to date, by the Psychiatric Genomics Consortium, further shows that the burden is enriched for genes associated with synaptic function and that deletions assert greater effect than duplications [12]. Despite strong evidence that CNVs are risk factors for schizophrenia and other developmental disorders, the mechanisms by which CNVs lead to disease onset remain unclear.

Endophenotypes are biomarkers that characterise illnesses and indicate genetic liability, as intermediate steps on the pathway from genes to disease [19, 20]. Cognitive function is one such endophenotype for schizophrenia and extensive literature shows that individuals with schizophrenia display reduced performance across a range of tests of specific and general cognition [21, 22]. This is not simply due to the effects of antipsychotic medication [23], and nor is it just an epiphenomenon of the symptoms of schizophrenia; cognition is impaired before illness onset [24, 25] as well as amongst the unaffected relatives of patients with schizophrenia [26–28]. A recent genome-wide association study with more than 269,000 samples shows a bidirectional effect with intelligence having a strong protective effect towards schizophrenia risk, and a smaller reverse effect, with schizophrenia predisposing to impaired cognitive functioning [29].

IQ and general cognitive ability are heritable [30–33]; however despite the identification of 205 loci affecting over 1000 genes associated with intelligence [29], they only explain ~5% of the inter-individual variability in intelligence. Part of the unexplained heritability of intelligence could be attributable to copy number variants. Many CNVs affect genes involved in neurodevelopment [15, 34, 35], providing a mechanism by which specific CNVs and CNV burden could affect cognition.

There is evidence linking several specific CNVs with schizophrenia, other neurodevelopmental disorders, educational attainment [36, 37] and with impaired cognition [38–41]. Furthermore, Stefansson et al. [42] showed that healthy carriers of any of 26 neuropsychiatric CNVs collectively performed at an intermediate level between healthy non-carriers and schizophrenia patients in several cognitive tests. This indicates that, while the risk CNVs may not have full penetrance for disease, most carriers will exhibit some degree of phenotypic change such as impaired cognition. A large study on the UK Biobank further supports this effect of neuropsychiatric CNVs impairing cognition in healthy carriers [37].

While the detrimental effects of specific schizophrenia-associated CNVs on cognition are well characterised, the influence of CNV burden on cognition is less clear. Some evidence, both in clinical samples and healthy populations, suggests that increased CNV burden is associated with lower IQ [30, 31, 43, 44], while other studies have failed to find this association [35, 45–47]. Until now, few studies have reported the effects of schizophrenia-associated CNVs or CNV burden on specific cognitive abilities [37, 42, 48].

Firstly, we conducted a systematic review and meta-analysis of the literature examining the relationship between CNV burden and general cognitive ability. We then present data from a new family study from the Psychosis Endophenotypes International Consortium (PEIC) [49] investigating the influence of CNVs (both burden and loci) on cognitive endophenotypes for schizophrenia [27, 50].

## Methods

### Meta-analysis of published association studies of CNVs and general IQ

We conducted a literature search using the databases Pubmed, Medline, and PsychINFO using the following search terms: “(CNV* OR copy number OR copy-number) AND (IQ OR intelligence OR cogniti*)”. The time window included any papers published before 1st April 2019. The reference and citations lists of relevant papers were examined to identify other relevant papers. We imposed no restriction on participant age, geographical location, or article language.

In addition to IQ, we included papers examining other measures of general intelligence as they are thought to be closely linked [51]. Papers investigating both patient and healthy populations were included. Where different papers used the same sample of participants, only the study with the most relevant phenotype was included. If multiple measures of intelligence were included (for example see references [47, 52]) the one deemed closest to the other studies was used for the meta-analysis. Similarly, if measures for both common and rare CNV-burden were reported [52] we included the latter for the meta-analysis.

Titles and abstracts of all relevant papers were screened to assess whether they met the inclusion criteria. Where necessary, we contacted the authors to request additional information needed to include the study in the meta-analysis. Supplementary Table 1 shows the data extracted from papers.

#### Meta-analysis of the literature

A random effects meta-analysis was conducted using StatsDirect version 3.0 [53] to calculate an overall estimate of the correlation between CNV burden and IQ for the included studies. Where primary studies reported Spearman’s correlations or standardised coefficients they were converted to Pearson’s correlation coefficients to ensure the studies were as comparable as possible [54, 55]. A random effects meta-analysis was chosen due to the variability in methods of the included studies (including different participant samples and inclusion criteria for CNVs). Statistical heterogeneity was measured using Cochran’s Q statistic.

### CNV analysis of the Psychosis Endophenotypes International Consortium sample

The initial dataset (prior to quality control) included 5597 participants from the PEIC family study [49], including people with schizophrenia, bipolar disorder with psychotic symptoms and other forms of psychosis, their unaffected relatives and unrelated controls. Participants were of European ancestry and assessments were conducted at nine centres: Amsterdam, Edinburgh, Groningen, London, Maastricht, Munich, Pamplona, Perth and Utrecht (see Supplementary Table 2 for further detail). Participants were recruited through clinical teams, voluntary organisations and press advertisements, and contributed both genetic data and cognitive performance measures [49]. All participants provided written informed consent and the study was approved by the respective ethical committees at each of the participating centres. Details of diagnostic classifications can be found in the Supplementary Methods.

#### Genotyping and quality control

DNA was obtained from blood for all participants and sent to the Wellcome Trust Sanger Institute (Cambridge, United Kingdom). Samples were genotyped with the Human SNP Array 6.0 at the Affymetrix Services Laboratory (www.affymetrix.com). We applied standard quality control procedures as described in the Supplementary Methods and in Bramon et al. [49]. CNVs were identified using PennCNV [56] and Affymetrix Power Tools, following the PennCNV-Affy protocol to calculate log R ratio (LRR) and B-allele frequency (BAF). Standard PennCNV settings were used and data were adjusted for genomic waves [57] using Affymetrix 6.0 GC-model file.

Individual-based quality control for the CNVs was performed using statistics calculated with PennCNV: quality control thresholds were determined based on inspection of the frequency distributions of the BAF-drift, LRR-standard deviation, and number of CNVs per participant, respectively. Individuals with either BAF-drift of >0.01, LRR-standard deviation of >0.5 or more than 300 CNV calls were removed. CNV-level quality control was performed by excluding CNVs with ten or fewer SNPs and by iteratively merging adjacent calls together if the length between calls was <20% of the combined length. Calls made by PennCNV in the pseudo-autosomal regions of the X-chromosome (10,000–2,781,479 bp and 155,701,382–156,030,895 bp, hg18) were excluded.

All CNVs included in the analysis were visually inspected by two researchers blind to clinical data using an in-house script to visualise BAF and LRR patterns. A consensus decision on inclusion was made between two researchers, both blind to clinical data, based on the comparison of the observed LRR and BAF of the affected region with the expected for a CNV with the given copy state. PennCNV frequently made CNV predictions that did not fit with the expected allelic and/or intensity pattern of the given copy state and thus 72% of CNV calls were discarded.

#### CNV burden analysis

Only rare (<1% frequency in the sample) and large (>200 kb) CNVs were included in the CNV burden. Frequency of CNVs were determined by identifying common CNV loci, through independent mapping of start and stop positions of all CNVs. CNVs whose start positions were within 300,000 bp of each other, where the stop position was also within a 300,000 bp bin were considered to be the same loci (see Supplementary Fig. 1 for details). In addition to calculations using total length of CNVs, we also measured burden as numbers of genes affected, as described in Marshall et al. [12]. We did this by annotating CNVs with RefSeq genes (hg18), including genes where at least one base pair of an exon overlapped with the CNV and adding up all unique genes affected in each individual. Total burden, and deletion and duplication burdens were analysed separately.

#### Analysis of schizophrenia-associated CNVs

We searched for carriers of 27 CNVs with good evidence of an association with schizophrenia as described by Marshall et al. [12], Kirov et al. [6] and Stefansson et al. [42] (see Supplementary Table 3). For the analysis of schizophrenia-associated CNVs we considered all samples prior to any sample/CNV-level quality control and performed visual inspection of all samples with CNV calls that overlapped with >10% of a schizophrenia-associated locus. Samples identified with true schizophrenia-associated CNVs by consensus of two researchers blind to clinical data, were included in the analysis even if they failed sample/CNV-level quality control. For 2p16 deletions, all CNV calls overlapping the region were inspected, and participants with validated CNVs affecting exons of the causative gene NRXN1 [58] were identified as carriers.

#### Cognitive measures

Cognitive measures collected from participants included block design [59, 60] (a test of perceptual reasoning), the combined digit span (measuring attention and working memory), and the Rey Auditory Verbal Learning Task (RAVLT) immediate and delayed recall (measuring short and long-term verbal memory, respectively). As different versions of these tests were used across centres, participants’ raw scores were converted into percentages by dividing each participant’s score by the maximum achievable score and multiplying by 100. Supplementary Table 2 details the number of participants for each cognitive measure.

#### Kinship matrix

The kinship coefficient is a probabilistic estimate that a random gene from a subject is “identical by descent” to a gene in the same locus from another subject. For “*n*” subjects, these probabilities can be assembled in an *n* × *n* “kinship matrix”, which can be used to model the covariance (or “relatedness”) between individuals and the population structure in a dataset. A kinship matrix based on a LD-pruned set of SNPs (102,112 SNPs selected with pruning parameters: *r*^2^ = 0.2; window = 1000 kb) was generated using LDAK [61] and added as a random effect to the linear and logistic mixed model regressions.

#### Statistical analysis

Firstly, the CNV burden data were not normally distributed showing clear zero inflation as expected. Therefore we performed a Wilcoxon rank sum test to compare the CNV burden of individuals with and without cognitive data available.

Secondly, the association between the CNV measures and clinical group was investigated. For this analysis, we performed mixed effects logistic regressions with disease status as outcome (either patients versus controls or relatives versus controls), age, gender and study centre as fixed effects and the kinship matrix as a random effect.

Thirdly, the relationships between known schizophrenia-associated CNVs and quantitative cognitive measures were examined using linear mixed models. The outcome variable was the cognitive measure and the predictor was the participants’ carrier status of schizophrenia-associated CNVs (carriers versus non-carriers). Given that schizophrenia-associated CNVs are very rare, with frequencies ranging from 0.01 to 0.3% [6, 42], only a combined analysis including several such CNVs was feasible. The analysis for a particular cognitive measure was only performed if data from at least ten carriers of schizophrenia-associated CNVs with cognitive measures were available. Finally, we also used linear mixed models to investigate the association between cognitive measures and CNV burden. Age, gender, clinical group (patient, relative and control), study centre and a kinship matrix were included as covariates in all linear mixed models. Analyses were performed using R version 3.5.0 [62]. All mixed model regressions including the kinship matrix as a random effect were performed using the lme4qtl R package [63].

As is standard practice in genetic association studies, we adjusted the significance threshold for multiple testing. We used two different analysis approaches depending on the whether the outcome variable was quantitative or categorical. Firstly, for the linear mixed models, we tested the correlations between all the cognitive outcomes and divided the significance threshold (0.05) by the effective number of traits, as per standard method [64, 65]. We investigated four cognitive measures: digit span, block design, RAVLT immediate, RAVLT delayed. These measures were strongly correlated [20], particularly, the last two (0.79), which was reflected by a calculation of the eigenvalues for the correlation matrix, which identified the number of effective traits as three. Secondly, for the mixed effects logistic regression analyses investigating categorical clinical group as outcomes, the number of effective traits were two, since we performed separate logistic regressions comparing cases versus controls and relatives versus controls. We present all uncorrected *p* values throughout the paper. However, interpretation of what constitute significant findings was based exclusively on the multiple-testing adjusted *p* value thresholds of 0.017 (0.05/3 effective traits) for cognition and of 0.025 (0.05/2 effective traits) for clinical group outcomes. All tests performed were two sided and the R-code used is available upon request from the corresponding author.

## Results

### Findings from the systematic review and meta-analysis of the literature

The literature search returned 905 results. Screening of titles and abstracts revealed 13 papers that were assessed for eligibility. See PRISMA diagram and details on the literature search in Supplementary Fig. 2. Eleven primary papers of similar quality were found to examine the association between CNV burden and intelligence [30, 31, 35, 43–47, 66, 67] (Supplementary Tables 1 and 4).

**Fig. 1:**
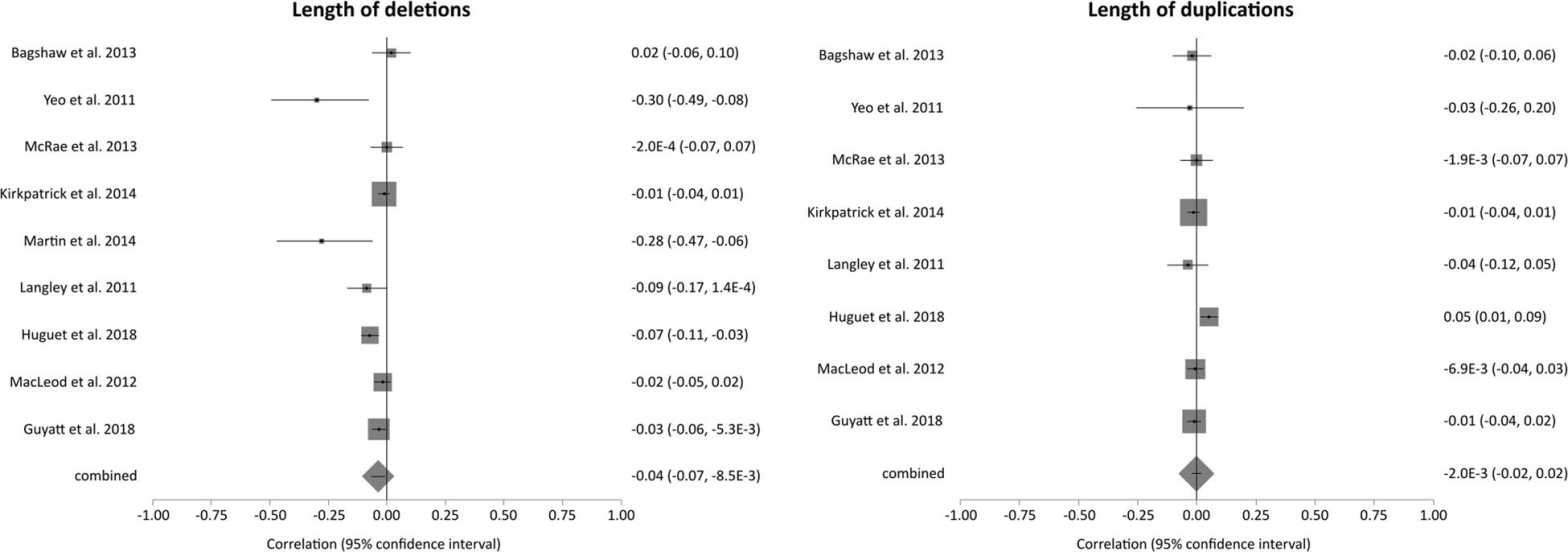
Forest plots for the meta-analyses investigating length of deletions (*N* = 18,658) and length of duplications (*N* = 18,580). For additional plots see Supplementary Fig. 3.

Ten studies, with a total of 18,847 participants, provided the required data to conduct a meta-analysis and were included in the random effects meta-analysis [30, 35, 43–45, 66, 67]. Forest plots for analyses of length of deletions (*N* = 18,658) and length of duplications (*N* = 18,580) are displayed in Fig. 1, additional forest plots can be found in Supplementary Fig. 3.

None of the meta-analyses showed evidence for an association between their measure of CNV burden and IQ. The pooled correlations between IQ and length of deletions or length of duplications were −0.04 (CI = −0.07, −0.01) and −0.002 (CI = −0.02, 0.02) respectively. Cochran’s *Q*-statistic revealed evidence for between-study heterogeneity in the correlation between length of deletions and IQ (*χ*^2^ = 21.56, *P* = 0.0058) and number of deletions and IQ (*χ*^2^ = 33.77, *P* < 0.0001), number of duplications (*χ*^2^ = 11.06, *P* = 0.0114). There was no evidence for study heterogeneity for the other measures.

One study included in the systematic review did not provide data suitable for the meta-analysis. However, its findings are consistent with the meta-analysis since Van Scheltinga et al. [46] found no association between their measures of CNV burden and IQ.

### Results from the Psychosis Endophenotypes International Consortium sample

The full sample consisted of 5597 participants. One thousand, three hundred three participants had CNV data failing quality control due to one or more of the following reasons: 1107 participants had more than 300 CNVs, 478 had BAF drift of >0.01 and 400 had Log R ratio standard deviation of >0.5.

In our study, 77% of samples passed stringent quality control criteria for CNV calling. This call rate is comparable to other CNV studies as exemplified by the latest Psychiatric Genomics Consortium CNV large mega-analysis reporting an overall 72% call rate across 43 primary studies [12]. The challenges with CNV calling from SNP microarrays, are well known [68], and is to a large extent technical in origin. SNP microarrays were not originally designed for CNV detection, and although it is possible as demonstrated by numerus publications, CNV detection from SNP arrays is sensitive to quality issues especially with regards to the intensity measures captured by the probes on the assay [68].

The 23% of samples excluded on quality control grounds did not differ significantly from those included in the study on key parameters including clinical group distribution and sex. The only significant difference was in age, where the excluded samples on average were 5 years younger (see full details in Supplementary Table 5). Age is included as a covariate in all analysis.

Of the 4294 participants who passed quality control, 3426 were included in the CNV burden analysis as they had at least one cognitive measure and full information on the included covariates. There were no significant differences in CNV burden measures between samples with and without cognitive data available (see Supplementary Table 6). An additional two participants that failed the CNV burden quality control were identified from two independent blind visual inspections as carriers of schizophrenia-associated CNVs, and included in that analysis (*N* = 3428). This sample included 769 patients with psychotic disorders (576 with schizophrenia (74.9%), 89 with bipolar disorder (11.6%) and 104 with other psychoses (13.5%), 646 unaffected relatives and 2013 healthy controls (see Table 1).

**Table 1:** Sample description.

Characteristic		CNV carrier (*N*)	Non-carrier (*N*)	Total (*N*)	CNV carrier (%)	
Clinical group						
Controls		16	1997	2013	0.8%	
Relatives		2	644	646	0.3%	
Patients		11	758	769	1.5%	
Centre						
Munich		3	949	952	0.3%	
Perth		6	548	554	1.1%	
London		2	478	480	0.5%	
Maastricht		4	395	399	1.0%	
Amsterdam		4	329	333	1.2%	
Utrecht		5	304	309	1.6%	
Groningen		5	310	315	1.6%	
Pamplona		0	44	44	0.0%	
Edinburgh		0	42	42	0.0%	
Sex						
Males		16	1743	1759	0.9%	
Females		13	1656	1669	0.8%	
**Characteristic**	**CNV carrier (*N*)**	**CNV carrier (mean/SD)**	**Non-carrier (*N*)**	**Non-carrier (mean/SD)**	**Total (*N*)**	**Total (mean/SD)**
Age	29	39.9 (15.6)	3399	43.7 (15.9)	3428	43.7 (15.9)
Cognitive performance						
Block design	21	50.6 (24.9)	2726	57.4 (23.6)	2747	57.4 (42.0)
Digit span	5	60.5 (4.5)	1265	50.7 (15.1)	1270	50.7 (9.4)
RAVLT immediate Recall	24	46.5 (16.6)	1882	54.7 (14.5)	1906	54.6 (14.9)
RAVLT delayed recall	24	14.6 (7.8)	1865	17.7 (6.9)	1889	17.7 (6.6)

Demographic information for participants with data on schizophrenia-associated CNVs. Two participants with schizophrenia-associated CNV were identified in the samples failing QC for CNV burden, these were included only in the analysis of schizophrenia-associated CNVs; thus giving a sample of 3428 participants for the analysis of schizophrenia-associated CNVs and 3426 for the analysis of CNV burden. CNV carriers refer to individuals who were identified as carrying a known schizophrenia-associated CNV (see Supplementary Table 3).

*RAVLT* Rey Auditory Verbal Learning Test, *SD* standard deviation.

In our sample, we identified 29 participants who carried one schizophrenia-associated CNV each (see loci in Supplementary Table 3). Table 2 shows the analyses of schizophrenia-associated CNVs and cognition, adjusted for age, gender, clinical group, centre and genetic relatedness. We found evidence of an association between schizophrenia-associated CNVs and RAVLT idiate (regression coefficient = −8.0, 95% CI = −13.3 to −2.6, *P* = 0.0036) and delayed (regression coefficient = −3.3, 95% CI = −5.8, −0.7, *P* = 0.0115) recall. This indicates that participants with a schizophrenia-associated CNV had a mean RAVLT immediate recall score that was 8.0% lower than non-carriers, as well as a mean RAVLT delayed recall score that was 3.3% lower. We also found suggestive evidence that carriers of a schizophrenia-associated CNV had poorer scores for block design than non-carriers (mean difference = −10.0, 95% CI = −19.2 to −0.9, *P* = 0.031) but only at the uncorrected level of significance. As a sensitivity analysis we performed the same associations using only patients with a schizophrenia diagnosis, their relatives and healthy controls, see Supplementary Table 7. In that analysis the association between CNV carrier status and RAVLT immediate recall remained significant (*P* = 0.004), and we observed a weaker association with RAVLT delayed recall score at the uncorrected significance level (*P* = 0.025).

**Table 2:** Associations between schizophrenia-associated CNVs and CNV burden with cognition.

Predictor	Cognitive measure	Participants (CNV Carrier)	Regression coefficient	95% CI	Sig.
Schizophrenia-associated CNVs	Block design	2747 (21)	−10.1	−19.2, −0.9	0.031
	Digit span	–	–	–	–
	RAVLT immediate Recall	1906 (24)	−8.0	−13.3, −2.6	**0.0036**
	RAVLT delayed recall	1889 (24)	−3.3	−5.8, −0.7	**0.0115**
Genes affected by all CNVs (burden)	Block design	2747	−0.1	−0.3, 0.1	0.343
	Digit span	1270	0.03	−0.2, 0.2	0.775
	RAVLT immediate recall	1906	−0.03	−0.2, 0.1	0.634
	RAVLT delayed recall	1889	−0.01	−0.1, 0.1	0.854
Genes affected by deletions (burden)	Block design	2747	−0.4	−0.8, 0.01	0.056
	Digit span	1270	−0.1	−0.6, 0.3	0.544
	RAVLT immediate recall	1906	−0.2	−0.5, −0.1	0.0119
	RAVLT delayed recall	1889	−0.1	−0.2, −0.01	0.0661
Genes affected by duplications (burden)	Block design	2747	−0.001	−0.2, 0.2	0.992
	Digit span	1270	0.06	−0.1, 0.3	0.564
	RAVLT immediate recall	1906	0.02	−0.1, 0.2	0.774
	RAVLT delayed recall	1889	0.03	−0.04, 0.1	0.412

Figures in bold are below the multiple testing adjusted *p*-value threshold of 0.017.

Associations between known schizophrenia-associated CNVs and CNV burden with cognitive performance. For the schizophrenia-associated CNV analysis digit span was not examined as fewer than ten CNV carriers had available data. CNV burden was measured as number of genes affected by CNVs larger than >200 kb, with <1% frequency. All analyses are adjusted for the covariates age, sex, clinical group, centre and genetic relatedness (kinship matrix).

Our CNV burden analysis showed that the mean total length of DNA affected by deletions and duplications was 117.8 and 219.1 kb, respectively and on average 1.8 genes per participant were affected by these CNVs. We did not find evidence for an increased total CNV burden measured as length of DNA affected by CNVs, or as number of genes affected by CNVs, in people with psychosis compared to healthy controls. Since Marshall et al. [12] found that the association between CNV burden and schizophrenia was more significant when indexed as number of genes affected, we focused our subsequent analyses on this measure. For analyses using CNV length see Supplementary Tables 8 and 9.

We found no evidence for an association between the four cognitive measures and any of the three CNV burden measures, see Table 2. Stratified analyses were also conducted by group (Supplementary Table 10), which showed an association between schizophrenia-associated CNVs and RAVLT immediate recall (regression coefficient = −11.8, 95% CI = −20.2 to −3.4, *P* = 0.006), and a weaker association with the RAVLT delayed recall (*P* = 0.02) in the patient group but only at the uncorrected level of significance. There was no other evidence for an association between the various cognitive measures and burden when examining the groups separately.

The mixed effects logistic regression suggests that there was no evidence in the difference of having a schizophrenia-associated CNV amongst patients, relatives and controls. Similarly for CNV burden, the number of genes affected by large CNVs did not differ between the three clinical groups. See Supplementary Table 11 for details of CNV comparisons between clinical groups. Cognitive performance was impaired in patients compared with controls for all variables examined, as expected, and was worse in relatives than controls for block design and digit span. See Supplementary Table 12 for adjusted analyses.

As a follow-up analysis we examined five loci found to protect carriers from developing schizophrenia [12, 69]. We identified 41 carriers (22q11.21.dup (*N* = 1), 7q11.21.del (*N* = 21), 7q11.21.dup (*N* = 7), 13q12.11.dup (*N* = 7) and Xq28.dup (*N* = 5)), but found no significant association between performance in cognitive tests and carrier status, or in number of carriers between patients, relatives or controls.

## Discussion

This study aimed to investigate: (1) the influence of CNV burden on general cognitive ability (IQ) based on a systematic review and meta-analysis of the literature and (2) the influence of schizophrenia-associated CNVs and CNV burden on specific cognitive skills in our family study from the PEIC.

The meta-analysis of published studies found no associations between any CNV burden measures and overall IQ. The PEIC sample revealed that carriers of specific schizophrenia-associated CNVs had clear impairments in immediate and delayed verbal recall. Verbal memory performance has been found to index cortical thinning in medial temporal and prefrontal regions in schizophrenia [70, 71] and has been found to be a cognitive predictor of outcome in schizophrenia and first episode psychosis [72, 73] supporting its role as a plausible endophenotype in psychosis. We also found suggestive evidence that carriers of schizophrenia-associated CNVs perform worse in block design although further investigation is needed to verify the link between schizophrenia-associated CNVs and this and similar measures of perceptual reasoning.

To the best of our knowledge, this is the first and only meta-analysis investigating the associations between CNV burden and IQ. We found ten relevant studies with a total of 18,847 participants. Evidence suggests that larger and rarer CNVs are more likely to be pathogenic [17]. Therefore, we hypothesised that the larger and rarer CNVs would have greater effects on intelligence. However, the primary studies included in our systematic review and meta-analysis did not always follow this pattern. Indeed the four studies, out of nine, that did find association with deletion length and intelligence included CNVs down to 100 kb or smaller. Two of these studies reported on fewer than 80 participants each. The two remaining studies [52, 67] were among the largest available and performed a rigorous quality control. They both found evidence that all of their rare deletion burden measures (CNV length and number of genes affected) resulted in lower IQ. We believe that at least part of the reason why none of the meta-analyses we performed found association between any of the measures of CNV burden and intelligence, is due to the heterogeneity in the methodology and CNV criteria of the studies conducted in this area, and it seems the field is still in need of a more consistent and stringent way to analyse CNV burden.

Along with Gialluisi et al. [48] our study is one of the first to report on associations between measures of large and rare CNV burden and performance on specific cognitive tests, and to our knowledge the first to examine this in both patients with psychosis and their unaffected relatives. The main limitation of our family study was the modest sample size, which limited our power to detect very small effects. CNV detection is known to be prone to false positive calls [74, 75], therefore a strength is the thoroughness taken in calling CNVs, as all calls included in our burden analysis were visually inspected by two researchers blind to all clinical data in order to ensure their accuracy. We discarded 72% of the CNVs predicted by PennCNV after visual inspection, which shows the importance of such checks. In our sample, we found no evidence of selection bias in relation to quality control or to availability of cognitive data. Thus, the percentage of individuals passing CNV quality control was similar between patients, relatives and controls and comparable to that of Marshall et al. [12]. Furthermore, the CNV burden did not differ between individuals with and without cognitive data available.

Our findings suggest that CNVs have greater effects on specific aspects of cognition rather than on general intelligence. Our family study investigated the burden of large (>200 kb), rare (<1% frequency) CNVs, which in general are expected to have greater phenotypic effects [5, 12, 76] than the smaller and more frequent CNVs included in many of the studies in the meta-analysis. Furthermore, the genetic contribution to IQ increases with age [77, 78], and in the meta-analysis there was substantial diversity in ages and clinical group examined across the studies. Finally, the relationship between CNV burden and intelligence may vary substantially with clinical status. Indeed, the majority of studies of the meta-analysis that included patients with neuropsychiatric conditions [30, 31, 44, 66] found associations between at least one measure of CNV burden and intelligence, whereas the majority of studies examining healthy participants [35, 45, 47] did not. However, our family study included 22.5% of participants with psychosis, compared to 0.8% participants in the meta-analysis, and still did not find an association with any burden measures.

Current available studies, including ours, may still be underpowered to detect a small effect of CNV burden on cognition. Furthermore, it is also important to consider the crudity of the current CNV burden measures defined by size and frequency criteria. As our understanding of the phenotypic effects of CNV improves, more specific burden measures targeting neurodevelopment and brain diseases will emerge. Limited power in our PEIC family study is likely to explain why we did not replicate the association between poorer digit-span and schizophrenia-associated CNVs, as reported by Kendall et al. [37] in the much larger UK Biobank study.

The RAVLT immediate recall and delayed recall, which measures working memory and long-term memory respectively, are robust endophenotypes of schizophrenia [27]. Thus, as hypothesised, we found they were impaired amongst the carriers of known schizophrenia-associated CNVs in the PEIC family based sample. The existing literature [37, 67, 79] as well as our data provide consistent evidence that the carriers of specific CNVs that increase schizophrenia risk have cognitive impairments. It is widely agreed that a better understanding of the genetics of psychosis is essential for developing new diagnostic and therapeutic interventions. Animal and cellular models will provide essential evidence to understand the mechanisms of the implicated genetic loci, but are only available for a few CNVs. Studying endophenotypes in the human in vivo is non-invasive and one of the best tools available to elucidate the role and mechanisms of genetic variants that increase the risk of developing neuropsychiatric disorders.

## Supplementary information

Supplementary material
