# Supplementary material for: Genetic copy number variants, cognition and psychosis: a meta-analysis and a family study
Source: Mol Psychiatry. 2020 Jul 27;26(9):5307–19. doi: 10.1038/s41380-020-0820-7 (PMC8589646; doi:10.1038/s41380-020-0820-7)
Supplement: Supplementary file 1 — Supplementary material [file 41380_2020_820_MOESM1_ESM.docx]

**Supplementary material**

[Supplementary methods: 2](#_Toc9347479)

[Phenotypes 2](#_Toc9347480)

[Genotyping 2](#_Toc9347481)

[DNA Sample Preparation 2](#_Toc9347482)

[Genotyping Methodology and Quality Control 2](#_Toc9347483)

[Data Quality Control 3](#_Toc9347484)

[Supplementary Tables 4](#_Toc9347485)

[Supplementary Table 1: Description of primary studies included in systematic review and meta-analysis 4](#_Toc9347486)

[Supplementary Table 2: Participants from each centre with cognitive measures 8](#_Toc9347487)

[Supplementary Table 3: Carriers of 27 schizophrenia-associated CNVs loci in the PEIC sample 9](#_Toc9347488)

[Supplementary Table 4: Association between the CNV measures and clinical group 10](#_Toc9347489)

[Supplementary Table 5: Assessments of study quality of primary studies 11](#_Toc9347490)

[Supplementary table 6: Comparisons between the sub-samples passing and failing QC 14](#_Toc9347491)

[Supplementary table 7: CNV burden in samples with and without cognitive data. 14](#_Toc9347492)

[Supplementary Table 8: Associations of clinical group and cognition, only including patients with a schizophrenia diagnosis 15](#_Toc9347493)

[Supplementary Table 9: Associations of clinical group with CNV burden measured as length 15](#_Toc9347494)

[Supplementary Table 10: Associations of CNV burden measured as length with cognitive performance 16](#_Toc9347495)

[Supplementary Table 11: Stratified subgroup analysis 17](#_Toc9347496)

[Supplementary Table 12: Association between clinical group and cognitive performance 18](#_Toc9347497)

[Supplementary Figures 19](#_Toc9347498)

[Supplementary Figure 1: Identifying common CNV loci 19](#_Toc9347499)

[Supplementary Figure 2: PRISMA Diagram 20](#_Toc9347500)

[Screening 20](file:///S:\MHS_Bramon\Millie-Johan-Jasmine%20Paper\molPsych\resubmission_feb2019\02_Thygesen_etAl_cnv_and_cognition_supplement_21May19.docx#_Toc9347501)

[Included 20](file:///S:\MHS_Bramon\Millie-Johan-Jasmine%20Paper\molPsych\resubmission_feb2019\02_Thygesen_etAl_cnv_and_cognition_supplement_21May19.docx#_Toc9347502)

[Eligibility 20](file:///S:\MHS_Bramon\Millie-Johan-Jasmine%20Paper\molPsych\resubmission_feb2019\02_Thygesen_etAl_cnv_and_cognition_supplement_21May19.docx#_Toc9347503)

[Identification 20](file:///S:\MHS_Bramon\Millie-Johan-Jasmine%20Paper\molPsych\resubmission_feb2019\02_Thygesen_etAl_cnv_and_cognition_supplement_21May19.docx#_Toc9347504)

[Supplementary Figure 3: Additional meta-analysis forest-plots 21](#_Toc9347505)

[References 22](#_Toc9347506)

# Supplementary methods:

## Phenotypes

Relatives and controls were not excluded if they had a personal history of non-psychotic psychiatric disorders (such as depression or anxiety), provided they were well and off psychotropic medication at the time of testing and for the preceding 12 months.

To confirm or rule out a DSM-IV^1^ diagnosis, all participants underwent a structured clinical interview with either the Comprehensive Assessment of Symptoms and History^2^, the Structured Clinical Interview for DSM Disorders^3^, the Schedule for Affective Disorders and Schizophrenia^4^ or the Schedule for Clinical Assessment in Neuropsychiatry, Version 2.0^5^. Participants were excluded if they had a history of neurologic disease or a loss of consciousness due to a head injury.

For the neuropsychological assessment, the Wechsler Adult Intelligence Scale, revised version^6^ or third edition^7^, were administered to participants. Performance on two subtests was used for analyses: the combined forward and backward digit span (measuring attention and working memory) and block design (measuring spatial visualisation). The Rey Auditory Verbal Learning Test^8^, including both immediate and delayed recall (assessing short- and long-term verbal memory, respectively), was also administered. Higher scores on the cognitive tasks indicate better performance. Additional information on the methodology for each site contributing data is reported elsewhere^9–15^.

## Genotyping

Genotyping methods and quality control details are described in full in Bramon et al. 2014^16^ and below.

### DNA Sample Preparation

Genomic DNA obtained from blood for all participants was sent to the Wellcome Trust Sanger Institute, Cambridge, United Kingdom. Samples were processed in 96-well plate format and each plate carried a positive and a negative control. DNA concentrations were quantified using a PicoGreen assay (Invitrogen, Life Technologies, Grand Island, New York) and an aliquot assayed by agarose gel electrophoresis. A sample passed quality control if the original DNA concentration was at least 50 ng/mL and the DNA was not degraded.

### Genotyping Methodology and Quality Control

To track sample identity, 30 single nucleotide polymorphisms (SNPs) including sex chromosome markers were typed on the Sequenom platform before entry to the whole genome genotyping pipeline. Of the initial 6935 samples, 347 failed quality control due to degraded or insufficient DNA or incorrect sex classification. The remaining samples were sent for genotyping with the Genome-wide Human SNP Array 6.0 at the Affymetrix Services Lab (<http://www.affymetrix.com>).

### Data Quality Control

Genotype calling was conducted using the CHIAMO algorithm (^17,18^) modified for use with the Affymetrix 6.0 genotyping array. A total of 11,610 SNPs with a study-wide missing data rate over 5% were excluded. Another 26,858 SNPs with four or more Mendelian inheritance errors identified with PEDSTATS were removed^19^. Additional exclusion criteria were departure from Hardy-Weinberg equilibrium (p < 10^–6^) or minor allele frequency (MAF) <0.02 with 2,404 and 145,097 SNPs removed, respectively. A total of 38,895 SNPs from the X or Y chromosomes or mitochondrial DNA were also excluded from the analysis. Finally, 9,499 poorly genotyped SNPs were removed following visual inspection of the genotyping intensity plots in the program Evoker^20^.

214 samples were excluded with more than 2% missing data across all SNPs. Another 70 samples were excluded due to divergent genome-wide heterozygosity (inbreeding coefficients were F > 0.076 or F < -0.076 as estimated with PLINK^21^. Chromosomal sharing was inferred from a genome-wide subset of 71,677 SNPs and from each duplicate pair the sample with the most complete genotype data was kept. 70 duplicates and monozygotic twins were removed by excluding one of each pair of individuals showing identity by descent greater than 95%.

Initial analysis of the genotype data identified a high fraction of samples (approximately 30%), which showed poor signal-to-noise ratio in the genotyping assay. Because the experimental source of the problem was unclear and to ensure a robust set of genotype calls, these samples were removed from further analysis. The sample loss was randomly distributed across the three clinical groups (32% of patients, 30% of relatives and 30% of controls; χ^2^ (2 df) = 3.2; P = 0.20).

After SNP quality control and CNV quality control as described in method section, 5,597 individuals remained. The current study included a subset of this larger sample, comprising 3,428 individuals who also had the relevant phenotypic and genetic data available.

# **Supplementary Tables**

## **Supplementary Table 1:** Description of primary studies included in systematic review and meta-analysis

| **Study** | **Participants** | **Number of participants** | **Mean age (SD)** | **Measure of Intelligence/ Cog.** | **Genotyping Array** | **CNV Length & Freq.** | **CNV Measures** | **Statistical Analysis** | **Outcome** |
| --- | --- | --- | --- | --- | --- | --- | --- | --- | --- |
| Bagshaw et al. 2013^22^ | Population birth cohort from the Christchurch Health and Development Study. | 723 (567 with WISC-R Total IQ score) | Exact values not given. Participants assessed at ages 8-9, 13, 18, and 25. | IQ assessed with WISC-R age 8-9. TOSCA age 13. BWRT age 18. Measure of overall academic achievement age 25. | Illumina Human660W-Quad BeadChip array. | >2 markers.  <5% frequency. | CNV count,  duplication length, deletion length. | Pearson’s correlations. | No significant associations. |
| Yeo et al. 2011^23^ | Alcohol dependence. | 74 | 39.9 (9.2) | IQ calculated from vocabulary and matrix reasoning subtests of WASI-I. | Illumina Human1M-Duo BeadChip array. | No minimum length specified.  <5% frequency. | Duplication count, deletion count,  duplication length, deletion length. | Pearson’s correlations. Regressions controlling for sex, ethnicity, and interactions of CNV burden with sex and ethnicity. | Correlations: Negative association between deletion length and IQ (r = -0.30, P = 0.010). Non-significant trend towards association between deletion count and IQ (r = 0.21, P = 0.080).  Regressions: Negative association between deletion length and IQ, for CNVs of <5% (p <0.001), <3% (P = <0.001) and <1% (P = 0.013) frequency. |
| McRae et al. 2013^24^ | Unrelated healthy participants from the Genetics of twin cognition study. | 800 | 16.5 (1.2) | IQ calculated from short version of the MAB. | Illumina 610K SNP array. | >20 Kb.  <5% frequency. | CNV count, duplication count, deletion count, CNV length, duplication length, deletion length. | Pearson’s correlations. | No significant associations. |
| Kirkpatrick et al. 2014^25^ | Healthy participants from the Minnesota Twin Family Study (2 different aged twin cohorts and parents), and the Sibling Interaction and Behaviour Study (parents, siblings, adoptees, and step-parents). | 6199 (2879 parents, 1015 17yr old twins, 1777 11yr old twins, 358 siblings, 95 adoptees, 75 step-parents.) | Parents = 43.4 (5.5); 17yr old twins = 17.5 (0.5); 11yr old twins = 11.8 (0.4); siblings = 14.9 (1.9); adoptees = 15.3 (2.2); step-parents = 40.7 (7.5) | IQ calculated from abbreviated versions of WISC-R (for participants <16 yr) and WAIS-R (for participants >16yr). | Illumina Human660W-Quad BeadChip array. | >15 markers.  <5% frequency. | CNV count, duplication count, deletion count, CNV length, duplication length, deletion length, homozygous deletion count, homozygous deletion length. | Regressions controlling for sex, birth year, and population stratification. Reported Pearson’s correlations but not significance levels for correlations. | Regressions: Negative association between duplication length IQ (P = 0.016) (not significant after correction for multiple testing). Non-significant trend towards association between duplication count and IQ (beta = 0.074, P = 0.053). |
| Martin et al. 2014^26^ | Patients with schizophrenia from Australian subsample of Levinson et al. (2011) GWAS. | 78 | Information not given. | Full scale IQ, verbal IQ, and performance IQ calculated from WASI-I. Premorbid IQ estimated with NART. | Affymetrix Genome-wide Human SNP array 6.0. | >10 Kb.  <1% frequency. | Deletion length | Spearman’s correlations. | Negative association between deletion length and full scale IQ (r = -0.267, P = 0.018), verbal IQ (r = -0.303, P = 0.007), and premorbid IQ (r = -0.249, P = 0.029). |
| Langley et al. 2011^27^ | Children with ADHD. | Original study: 525.  Burden analysis: 520. | 10.6 (2.8) | IQ calculated from WISC. Participants aged <12 tested with the WORD (a test of reading ability). | Illumina Human660W-Quad BeadChip array. | Original study:  >500 Kb, <1% frequency. Burden analysis: >200 Kb, <1% frequency. | Original study: Participants with vs without at least one CNV.  Burden analysis: Deletion length, duplication length. | Original study: T-tests. Separate t-tests for full sample and sample with participants with ID removed.  Burden analysis: Spearman’s correlations. | Original study: With the full sample, carriers had lower IQ (P = 0.020) and reading ability (P = 0.020) than non-carriers. With participants with ID removed, CNV carriers had lower reading ability than non-carriers (P = 0.020), but not lower IQ. Burden analysis: non-significant trend  towards association between deletion length and IQ (r = 0.08, P = 0.063). |
| van Scheltinga et al. 2013^28^ | Patients with schizophrenia and healthy controls. | 672 (350 patients, 322 healthy controls.) | Patients with schizophrenia = 30.2 (9.0)  Healthy controls = 31.6 (11.9) | IQ calculated from 4/11 subtests of the WAIS-III or WAIS-IIIR. (block design, comprehension, vocabulary, picture arrangement). | Illumina HumanHap550 BeadChip array. | >9 markers.  No frequency limit specified. | Duplication count, deletion count, number of genes affected by duplications, number of genes affected by deletions. | ANOVA, using duplication/deletion count, no. of genes affected by duplications/deletions, and clinical group as factors, and IQ as dependent variable. Separate ANOVAs for deletions and duplications. | No significant associations between CNV measures and IQ. |
| MacLeod et al. 2012^29^ | Healthy elderly people from 4 cohorts: Lothian birth cohort 1921, Lothian birth cohort 1936, the Aberdeen birth cohort 1936, and participants from the Manchester age and cognitive performance research centre programme. | Participants with a crystallised intelligence score = 3210.  Participants with fluid intelligence score = 3133. | Mean ages for study sample not given.  Mean ages of cohorts:  Lothian 1921 = 79.1 (0.6), Lothian 1936 = 69.5 (0.8), Aberdeen 1936 = 64.6 (0.9), Manchester cohort = 65.6 (14.3) | Crystallised intelligence factor represented by NART or MHVT scores. Fluid intelligence factor derived from PCA of Moray house test, Raven's matrices, logical memory tests, verbal fluency tests, WASI-III, and RAVLT. (Different tests given to different cohorts).The 2 factors were corrected for age and sex. | Illumina610-Quad v1 chip array. | >500 Kb, 200-500kb and 100-200kb  <1% frequency. | CNV count, duplication count, deletion count, CNV length, duplication length, deletion length, number of genes affected by duplications, number of genes affected by deletions. | Regressions, adjusted for cohort.  T-tests compared the 2 intelligence factors between CNV carriers and non-carriers. | Regressions: no associations found between any of the CNV measures and either intelligence factor  T-tests: no difference in either intelligence factors between CNV carriers and non-carriers. |
| Yeo et al. 2013^30^ | Patients with schizophrenia and healthy controls. | 189 (79 patients, 110 controls) | Patients with schizophrenia = 35.0 (11.4)  Controls = 31.7 (10.9) | A 'general cognitive ability' factor was derived with a PCA from results of several tests, including reading ability, working memory, verbal abstraction, non-verbal reasoning, attention, visuo-motor skills, and executive skills. | Illumina Human  Omni1-quad BeadChip array | >500 bp.  <3% frequency. | Deletion count, deletion length. | Regressions, adjusted for clinical group, age, sex, ethnicity, and the interaction of deletion count/length with age and with clinical group. | Negative association between deletion count and general cognitive ability (beta = -0.19, p <0.001). |
| Huguet et al. 2018^31^ | Adolescents from the IMAGEN study and children and their parents from the Saguenay Youth Study (SYS) | 2711 | IMAGEN cohort = 14.5 years (0.4)  SYS cohort 610Kq Illumina =  14.5 years (1.9)*  SYS cohort Human Omni Express Version 12 =  14.9 months (1.8)*  *Average age only presented for the children | Performance IQ and Verbal IQ | IMAGEN cohort was genotyped using a combination of the Illumina 610Kq and 660Wq  SYS cohort was genotyped using the Illumina 601Kq and HumanOmniExpress BeadChip V12 | >50kb  <0.1% frequency in the database of genomic variants or a non-recurrent CNVs with the following characteristics: (i) DGV frequency < 0.1%; (ii) < 50% of the CNV is contained in regions present at > 1% in DGV8–10; (iii) seen only once in each cohort | Deletion length, duplication length, number of genes affected by deletions, number of genes affected by duplications, number of exonic deletions and number of exonic duplications | Regressions, adjusted for first six principal components, sex, age, array and familial relatedness | Negative association between length of rare deletions and PIQ (standardised beta coefficient = -0.07, p = 0.000561). There was also evidence for an associated between number of genes affected by rare deletions and PIQ (standardised beta coefficient = -0.06). No associations were found between rare duplications and PIQ for common deletions and duplications and either measure of IQ. |
| Guyatt et al. 2018^32^ | Children from the Avon Longitudinal Study of Parents and Children (ALSPAC) | 4576 with IQ, including 85 carriers of known pathogenic CNVs | Exact values not given, IQ was assessed at age 8 | IQ assessed with WISC at age 8 | Illumina HumanHap 55o-Quad | <1%  >100kb | Number of genes affected by deletions, number of genes affected by duplications, length of deletions and length of duplications | Regressions, adjusted for sex and first two principal components | IQ was associated with CNV burden: the SMD per gene affected by deletions was -0.019 (95% CI  -0.028 to -0.009, p = 2e-04) and the SMD per gene affected by duplications was -0.016 (95% CI -0.024 to -0.008, p = 1e-04). IQ was also associated with CNV length. For the total length of CNVs, the SMD per 100kb of deletions was -0.035 (95% CI -0.048 to -0.022, p = 2e-07) and the SMD per 100kb of duplications was -0.012 (95% CI -0.01 to -0.003, p = 0.008). |
| **Note:** Count refers to the number of CNVs of a given type, length refers to the number of bases affected by CNVs of a given type. CNV length/count refers to the length/count of all CNVs (both deletions and duplications).  **Abbreviations:** bp, Base pairs; Kb, kilo bases; WICS-R, Wechsler Intelligence Scale for Children – Revised; TOSCA, Test of Scholastic Abilities; BWRT, Burt Word Reading Test; WASI-I, Wechsler Adult Intelligence Scale – I; WASI-III, Wechsler Adult Intelligence Scale – III; WASI-IIIR, Wechsler Adult Intelligence Scale – III Revised; MAB, Multidimensional Aptitude Battery; GWAS, Genome-Wide Association Study; NART, National Adult Reading Test; ADHD, Attention Deficit Hyperactivity Disorder; WORD, Wechsler Objective Reading Dimensions ID, Intellectual Disability; ANOVA, Analysis of Variance; MHVT, Mill Hill Vocabulary Test; PCA, Principal Components Analysis; RAVLT, Rey Auditory Verbal Learning Test. | | | | | | | | | |

The 11 primary studies included in the systematic review and the sub-set of 10 contributing to the meta-analysis were diverse. Some studies included only healthy participants^22,24,25,29,31^, whereas others included clinical samples^23,26–28,30^. They also differed in sample size, age and gender distribution of participants. Nine of the papers examined the association between CNV burden and IQ, whereas two papers examined constructs other than ‘standard IQ’. MacLeod et al. 2012^29^ measured a ‘fluid intelligence’ and a ‘crystallised intelligence’ factor, and Yeo et al. 2013^30^ measured a ‘general cognitive ability’ factor. Both derived these factors from a principal components analysis of the results of several cognitive tests. Different measures of CNV burden were used, such as number or length of CNVs and examining duplication and deletions separate or together. Some studies specified a minimum length for a CNV to be included^22,24,26–32^, others did not^23^. Some studies only included CNVs with less than 1% frequency in their sample^26,27,29,31,32^, others used a 5% cut-off^22–25^. Six papers used correlations to investigate the association between CNV burden and IQ: two papers used Spearman’s correlations^26,27^ and four papers used Pearson’s correlations^22–25^. Additionally, five papers reported regression analyses^23,25,29–32^, one reported T-tests^27^, and one reported an analysis of variance^28^.

## Supplementary Table 2: Participants from each centre with cognitive measures

|  |  |  | **Cognitive Measures** | | | |
| --- | --- | --- | --- | --- | --- | --- |
| **Affiliation** | **City** | **Country** | **Block Design** | **Digit Span** | **RAVLT Immediate Recall** | **RAVLT Delayed Recall** |
| University of Edinburgh | Edinburgh | United Kingdom | 42 | 36 | 0 | 0 |
| GROUP Consortium (University of Amsterdam, University of Groningen, Maastricht University, University of Utrecht) | Amsterdam, Groningen, Maastricht, Utrecht | Netherlands | 1307 | 0 | 1302 | 1285 |
| Institute of Psychiatry, King's College London | London | United Kingdom | 446 | 238 | 6 | 6 |
| Ludwig-Maximilians, University of Munich | Munich | Germany | 952 | 952 | 0 | 0 |
| Fundacion Argibide, Pamplona | Pamplona | Spain | 0 | 44 | 44 | 44 |
| The University of Western Australia | Perth | Australia | 0 | 0 | 554 | 554 |
| **Total** |  |  | 2747 | 1270 | 1906 | 1889 |

RAVLT = the Rey Auditory Verbal Learning Task.

## Supplementary Table 3: Carriers of 27 schizophrenia-associated CNVs loci in the PEIC sample

| **Locus** | **Chromosome** | **Start Position(Hg18)** | **Stop Position(Hg18)** | **Size (Mb)** | **Gene of Interest** | **No. found** | | **Odds Ratio** | **Frequency in controls** | **Reference** |
| --- | --- | --- | --- | --- | --- | --- | --- | --- | --- | --- |
| 1q21.1.del | chr1 | 144800000 | 146326000 | 1.5 |  | | 3 | 3.8-8.1 | 0.02-0.07 | ^33–35^ |
| 1q21.1.dup | chr1 | 144800000 | 146326000 | 1.5 |  | | 0 | 2.9-4.2 | 0.03-0.07 | ^33–35^ |
| 2p25.3.dup | chr2 | 1733000 | 2204000 | 0.5 | MYT1L | | 2 | 15.7 |  | ^34^ |
| 2p16.del | chr2 | 49900000 | 51500000 | 1.6 | NRXN1 | | 2 | 10.7-14.4 | 0.014 | ^33–35^ |
| 3q29.del | chr3 | 197185549 | 198838373 | 1.7 | PAK2, DLG1 | | 0 | 18-63 | 0-0.001 | ^33–35^ |
| 7q11.23 | chr7 | 72380000 | 73780000 | 1.4 |  | | 0 | 16.1 | 0.004-0.28 | ^33,35^ |
| 7q36.3.del | chr7 | 158448321 | 158810016 | 0.4 | VIPR2 | | 0 | 3.5 | 0.029 | ^33^ |
| 7q36.3.dup | chr7 | 158448321 | 158810016 | 0.4 | VIPR2 | | 2 | 3.2-3.5 | 0.029 | ^33,34^ |
| 8q22.2 | chr8 | 100094670 | 100958984 | 0.9 | VPS13B | | 0 | 14.5 | 0.004 | ^33^ |
| 9p24.3.del | chr9 | 831690 | 959090 | 0.1 | DMRT1 | | 0 | 12.4 | 0.004 | ^33^ |
| 9p24.3.dup | chr9 | 831690 | 959090 | 0.1 | DMRT1 | | 0 | 12.4 | 0.004 | ^33^ |
| 15q11.2.del | chr15 | 20301000 | 20824174 | 0.5 | CYFIP1 | | 7 | 1.8-2.1 | 0.25-0.27 | ^33–35^ |
| 15q11.2-13.1.dup | chr15 | 20322358 | 26208861 | 5.9 |  | | 1 | 5.1 |  | ^34,35^ |
| 15q13.3.I.del | chr15 | 28723577 | 30303141 | 1.6 | CHRNA7 | | 1 | 4.7-15.6 | 0.009 | ^33–35^ |
| 15q13.3.II.del | chr15 | 29806023 | 30407419 | 0.6 | CHRNA7 | | 0 | 14.9 |  | ^34^ |
| 16p13.11.dup | chr16 | 14897345 | 16199484 | 1.3 | NTAN1, NDE1 | | 5 | 2-2.2 | 0.13 | ^34,35^ |
| 16p13.11.del | chr16 | 15032942 | 16199484 | 1.2 | NTAN1, NDE1 | | 0 | 1.9 |  | ^34^ |
| 16p12.1.del | chr16 | 21854731 | 22331199 | 0.5 |  | | 1 | 1.8 |  | ^34^ |
| 16p11.2.distal.del | chr16 | 28721599 | 28950951 | 0.2 |  | | 0 | 2.6-20.6 | 0.004-0.01 | ^33–35^ |
| 16p11.2.del | chr16 | 29502984 | 30100062 | 0.6 |  | | 2 | 0.5-0.9 | 0.04 | ^34,35^ |
| 16p11.2.dup | chr16 | 29531748 | 30105652 | 0.6 |  | | 1 | 8-9.4 | 0.03 | ^33–35^ |
| 17p12.del | chr17 | 14041754 | 15411904 | 1.4 |  | | 0 | 5.7 | 5.7 | ^34^ |
| 17q12.del | chr17 | 31889664 | 33323543 | 1.4 | HNF1B | | 0 | 4-9.5 | 0.005 | ^34,35^ |
| 17q12.dup | chr17 | 31889664 | 33323543 | 1.4 | HNF1B | | 0 | 2 |  | ^34^ |
| 22q11.21.large.del | chr22 | 17285281 | 19818855 | 2.5 |  | | 1 | 67.7 | 0.04 | ^33,35^ |
| 22q11.21.del | chr22 | 19063495 | 19795780 | 0.7 |  | | 1 | Inf |  | ^34^ |
| Xq28.distal.dup | chrX | 153800000 | 154225000 | 0.4 |  | | 0 | 0.35 | 0.18 | ^33^ |

The loci comprise all schizophrenia associated loci from Marshall et al. 2016^33^, Kirov et al. 2014^35^ and Stefansson et al. 2014^34^, excluding protective loci 22q11.21.dup, 7q11.21.del 7q11.21.dup, 13q12.11.dup, Xq28.dup. No. found indicate number of carriers found in the PEIC sample.

## Supplementary Table 4: Assessments of study quality of primary studies

Assessments of study quality for studies meeting the inclusion criteria for the meta-analysis, using the quality assessment tool for observational, cohort, and cross-sectional studies from the National Institute of Health^36^. Studies were assessed to be of similar quality. Two studies did not have uniformly-applied outcome measures for all participants^28,29^.

| **Criteria** | | **Bagshaw et al. 2013** | **Yeo et al. 2011** | **McRae et al. 2013** | **Kirkpatrick et al. 2014** | **Martin et al. 2014** | **Langley et al. 2011** | **van Scheltinga et al. 2013** | **MacLeod et al. 2012** | **Yeo et al. 2013** | **Huguet et al. 2018** | **Guyatt et al. 2018** |
| --- | --- | --- | --- | --- | --- | --- | --- | --- | --- | --- | --- | --- |
| 1 | Was the research question or objective in this paper clearly stated? | Yes | Yes | Yes | Yes | Yes | Yes | Yes | Yes | Yes | Yes | Yes |
| 2 | Was the study population clearly specified and defined? | Yes | Yes | Yes | Yes | Yes | Yes | Yes | Yes | Yes | Yes | Yes |
| 3 | Was the participation rate of eligible persons at least 50%? | NR | NR | NR | NR | NR | NR | NR | NR | NR | NR | NR |
| 4 | Were all the subjects selected or recruited from the same or similar populations (including the same time period)? Were inclusion and exclusion criteria for being in the study prespecified and applied uniformly to all participants? | Yes | Yes | Yes | No^4^ | Yes | No^5^ | No  (Point not deducted) | No^4^ | No^6^  (Point not deducted) | No^7^ | Yes |
| 5 | Was a sample size justification, power description, or variance and effect estimates provided? | No | No | No | No | No | No | No | No | No | No | No |
| 6^1^ | For the analyses in this paper, were the exposure(s) of interest measured prior to the outcome(s) being measured? | NA | NA | NA | NA | NA | NA | NA | NA | NA | NA | NA |
| 7^1^ | Was the timeframe sufficient so that one could reasonably expect to see an association between exposure and outcome if it existed? | NA | NA | NA | NA | NA | NA | NA | NA | NA | NA | NA |
| 8 | For exposures that can vary in amount or level, did the study examine different levels of the exposure as related to the outcome (e.g., categories of exposure, or exposure measured as a continuous variable)? | Yes | Yes | Yes | Yes | Yes | Original study: no. Burden analysis: yes. | Yes | Yes | Yes | Yes | Yes |
| 9 | Were the exposure measures (independent variables) clearly defined, valid, reliable, and implemented consistently across all study participants? | Yes | Yes | Yes | Yes | Yes | Yes | Yes | Yes | Yes | Yes | Yes |
| 10^1^ | Was the exposure(s) assessed more than once over time? | NA | NA | NA | NA | NA | NA | NA | NA | NA | NA | NA |
| 11 | Were the outcome measures (dependent variables) clearly defined, valid, reliable, and implemented consistently across all study participants? | Yes | Yes | Yes | Yes^8^ | Yes | Yes^9^ | No^10^ | No^11^ | Yes | No^12^ | Yes |
| 12 | Were the outcome assessors blinded to the exposure status of participants? | NR | NR | NR | NR | NR | NR | NR | NR | NR | NR | NR |
| 13^2^ | Was loss to follow-up after baseline 20% or less? | NA | NA | NA | NA | NA | NA | NA | NA | NA | NA | NA |
| 14^3^ | Were key potential confounding variables measured and adjusted statistically for their impact on the relationship between exposure(s) and outcome(s)? | No | No | No | No | No | No | No | No | No | No | No |
| Ratings: Yes, No, NA (not applicable), NR (not reported), CD (cannot determine). | | | | | | | | | | | | |
| ^1^ Criteria 6, 7, and 10 refer to the time-frame of assessing the exposure. All studies are marked NA for this because we know that CNVs are present and fixed at birth, therefore we know that the exposure (presence of CNVs) occurred before the outcome, so it doesn’t matter if the exposure was assessed before the outcome (criteria 6). Since CNVs are present at birth, we know that there will have been sufficient time for the association to have developed if it existed (criteria 7), and since CNVs are fixed and constant, there is no need to assess the exposure more than once over time (criteria 10). | | | | | | | | | | | | |
| ^2^ Criteria 13 is marked as NA for all studies because baseline and follow-up assessments were not necessary to measure the exposure (CNV burden) or outcome (IQ). | | | | | | | | | | | | |
| ^3^ Age, sex, ethnicity and education level were regarded to be the key potential confounding variables. | | | | | | | | | | |  |  |
| ^4^ Participants from cohorts of different ages were included in the study. | | | | | | | | | | |  |  |
| ^5^ Some participants had intellectual disability, whereas others did not. | | | | | | | | | | |  |  |
| ^6^ Both participants with schizophrenia and healthy controls were included. However, this was adjusted for in the analysis therefore a point was not deducted for this. | | | | | | | | | | |  |  |
| ^7^The study included adolescents from the IMAGEN study and children and parents from the Saguenay Youth Study. | | | | | | | | | | |  |  |
| ^8^ Two different versions of the IQ test were used: the WISC-R (Wechsler Intelligence Scale for Children - Revised) for participants aged <16, and the WAIS-R (Wechsler Adult Intelligence Scale - Revised) for participants aged >16. This is because it would not be appropriate to test participants aged <16 with an adult IQ test or participants aged >16 with a children's IQ test, therefore this was not considered to be inconsistent implementation of outcome measures across participants, so this criteria was marked as a ‘yes’. | | | | | | | | | | |  |  |
| ^9^ The WORD (Wechsler Objective Reading Dimensions) assessment package was only given to participants aged <12, therefore was not implemented uniformly across study participants. However, this is not the outcome that we were interested in for the meta-analysis. Our outcome of interest, IQ, was assessed uniformly across participants, therefore this criteria was marked as a 'yes'. | | | | | | | | | | |  |  |
| ^10^ Two different versions of the WAIS (Wechsler Adult Intelligence Scale) were used: the WAIS-III and the WAIS-IIIR (revised). | | | | | | | | | | |  |  |
| ^11^ Different outcome measures were given to the different cohorts included in the study. | | | | | | | | | | |  |  |
| ^12^ Two different versions of the IQ test were used: the WISC-IV edition was used in the IMAGEN cohort and the WISC-III was used for the SYS Cohort | | | | | | | | | | |  |  |

## **Supplementary table 5: Comparisons between the sub-samples passing and failing QC**

| **Parameter compared** | **Sub-sample passing QC**  **N=4,294** | **Sub-sample failing QC**  **N=1,303** | **Statistics** |
| --- | --- | --- | --- |
| Clinical group distribution | 55% patients | 49% patients | P-value = 1 (Chi-squared test) |
|  | 26% controls | 34% controls |  |
|  | 19% relatives | 17% relatives |  |
| Sex | 47.7% females | 46.1% females | P-value = 0.33 (Chi-squared test) |
| Mean age (SD) | 42.8 (15.6) years | 37.8 (15.0) years | P-value = 4.8×10^-20^ (t-test) |

## **Supplementary table 6: CNV burden in samples with and without cognitive data.**

| **Burden** | **No Cognition or covariates available (N = 874)**  **Median number of genes affected (Q1-Q3)** | **Study sample (N = 3,428)**  **Median number of genes affected (Q1-Q3)** | **Wilcoxon rank-sum test**  **p-value** |
| --- | --- | --- | --- |
| CNV total | 1 (0-6) | 0 (0-2) | 0.30 |
| CNV duplications | 0 (0-3) | 0 (0-0) | 0.27 |
| CNV deletions | 0 (0-0) | 0 (0-0) | 0.82 |

## **Supplementary Table 7:** Associations of clinical group and cognition, only including patients with a schizophrenia diagnosis

| **Predictor** | **Cognitive Measure** | **Participants (Associated CNV Carrier)** | **Regression Coefficient** | **95% CI** | **Sig.** |
| --- | --- | --- | --- | --- | --- |
|  |  |  |  |  |  |
| Schizophrenia-associated CNVs | Block Design | 2326 (18) | -9.787 | -19.4, -0.186 | 0.046 |
|  | RAVLT Immediate Recall | 1541(22) | -8.39 | -14.1, -2.72 | 0.004 |
|  | RAVLT Delayed Recall | 1524 (22) | -3.10 | -5.82, -0.385 | 0.025 |
|  |  |  |  |  |  |
| Genes affected by all CNVs | Block Design | 2324 | -0.117 | -0.323, 0.090 | 0.268 |
|  | Digit Span | 1188 | 0.026 | -0.158, 0.210 | 0.780 |
|  | RAVLT Immediate Recall | 1539 | -0.039 | -0.181, 0.103 | 0.592 |
|  | RAVLT Delayed Recall | 1522 | -0.025 | -0.093, 0.043 | 0.474 |
|  |  |  |  |  |  |
| Genes affected by deletions (deletion burden) | Block Design | 2324 | -0.440 | -0.851, -0.029 | 0.036 |
|  | Digit Span | 1188 | -0.198 | -0.662. 0.267 | 0.404 |
|  | RAVLT Immediate Recall | 1539 | -0.115 | -0.396, 0.166 | 0.423 |
|  | RAVLT Delayed Recall | 1522 | -0.082 | -0.216, 0.052 | 0.235 |
|  |  |  |  |  |  |
| Genes affected by duplications (duplication burden) | Block Design | 2324 | -0.008 | -0.245, 0.230 | 0.950 |
|  | Digit Span | 1188 | 0.066 | -0.131, 0.263 | 0.510 |
|  | RAVLT Immediate Recall | 1539 | -0.013 | -0.177, 0.152 | 0.881 |
|  | RAVLT Delayed Recall | 1522 | -0.005 | -0.084, 0.0073 | 0.895 |
| Associations between known schizophrenia-associated CNVs and CNV burden with cognitive performance. For the schizophrenia associated CNV analysis digit span was not examined as fewer than 10 CNV carriers had available data. CNV burden was measured as number of genes affected by CNVs larger than >200 Kb, with <1% frequency. All analyses are adjusted for the covariates age, sex, clinical group, centre and genetic relatedness (kinship matrix). | | | | | |

## **Supplementary Table 8:** Associations of clinical group with CNV burden measured as length

| **Predictor** | **Clinical group** | **Observations** | **Odds Ratio** | **95% CI** | **P-value** |
| --- | --- | --- | --- | --- | --- |
| Length of all CNVs (total burden) | Patient vs Control | 2780 | 1 | -1.0, 3.0 | 0.302 |
|  | Relative vs Control | 2658 | 1 | -1.0, 3.0 | 0.303 |
|  |  |  |  |  |  |
| Length of deletions (deletion burden) | Patient vs Control | 2780 | 1 | -1.0, 3.0 | 0.596 |
|  | Relative vs Control | 2658 | 1 | -1.0, 3.0 | 0.101 |
|  |  |  |  |  |  |
| Length of duplications (duplication burden) | Patient vs Control | 2780 | 1 | -1.0, 3.0 | 0.371 |
|  | Relative vs Control | 2658 | 1 | -1.0, 3.0 | 0.962 |
| All analyses were adjusted for gender, centre, age and genetic relatedness (kinship matrix). CNV burden was measured as the length of DNA affected by CNVs larger than >200 Kb, with <1% frequency. | | | | | |

## **Supplementary Table 9:** Associations of CNV burden measured as length with cognitive performance

| **Predictor** | **Cognitive Measure** | **Regression Coefficient** | **95% CI** | **Sig.** |
| --- | --- | --- | --- | --- |
|  |  |  |  |  |
| Length of all CNVs (total burden) | Block Design | 7.50E-04 | 1.88E-03, 3.73E+04 | 0.190 |
|  |  |  |  |  |
|  | Digit Span | -7.65E-05 | 1.2E-03, 1.10E+03 | 0.898 |
|  | RAVLT Immediate Recall | 9.24E-05 | 7.2E-04, 9.03E+04 | 0.823 |
|  | RAVLT Delayed Recall | 4.36E-05 | 3.42E-04, 4.29E+04 | 0.824 |
|  |  |  |  |  |
| Length of deletions (deletion burden) | Block Design | 1.79E-03 | 3.81E-03, 2.28E+04 | 0.082 |
|  |  |  |  |  |
|  | Digit Span | 4.00E-04 | 2.9E-03, 2.11E+03 | 0.754 |
|  | RVLT Immediate Recall | 1.12E-03 | 2.54E-03, 1.26E+03 | 0.121 |
|  | RAVLT Delayed Recall | 6.20E-04 | 1.29E-03, 5.47E-05 | 0.072 |
|  |  |  |  |  |
| Length of duplications (duplication burden) | Block Design | 2.90E-04 | 1.65E-03, 1.08E+03 | 0.680 |
|  |  |  |  |  |
|  | Digit Span | 1.38E-05 | 1.31E-03, 1.33E+03 | 0.984 |
|  | RAVLT Immediate Recall | 6.86E+04 | 3.0E-04, 1.67E+03 | 0.174 |
|  | RAVLT Delayed Recall | 3.71E+04 | 1.0E-04, 8.44E+03 | 0.123 |
| Associations between CNV burden and cognitive performance. CNV burden was measured as Kb base pairs affected by CNVs larger than 200kb, with <1% frequency. All analyses adjusted for the covariates age, sex, clinical group, centre and genetic relatedness (kinship matrix). | | | | |
|  |  |  |  |  |
|  |  |  |  |  |
|  |  |  |  |  |

## **Supplementary Table 10:** Stratified subgroup analysis

| **Predictor** | **Cognitive Measure** | **Group** | **Participants (Associated CNV Carrier)** | **Regression Coefficient** | **95% CI** | **Sig.** |
| --- | --- | --- | --- | --- | --- | --- |
|  |  |  |  |  |  |  |
| Schizophrenia-associated CNVs | Block Design | Patients | 420 (5) | -26.2 | -48.9, -3.51 | 0.024 |
|  |  | Relatives | 502 (2) | -16.5 | -47.0, 13.9 | 0.288 |
|  |  | Controls | 1825 (14) | -1.94 | -12.1, 8.26 | 0.709 |
|  | RAVLT Immediate Recall | Patients | 597 (10) | -11.8 | -20.2, -3.38 | 0.006 |
|  |  | Relatives | 516 (2) | 3.95 | -14.2, 22.1 | 0.669 |
|  |  | Controls | 793 (12) | -6.18 | -13.5, 1.14 | 0.098 |
|  | RAVLT Delayed Recall | Patients | 595 (10) | -4.64 | -8.54, -0.727 | 0.020 |
|  |  | Relatives | 513 (2) | -1.23 | -9.856, 7.40 | 0.781 |
|  |  | Controls | 781 (12) | -2.01 | -5.50, 1.48 | 0.259 |
|  |  |  |  |  |  |  |
| Genes affected by all CNVs | Block Design | Patients | 420 | 0.046 | -0.556, 0.648 | 0.881 |
|  |  | Relatives | 502 | -0.136 | -0.648, 0.376 | 0.603 |
|  |  | Controls | 1825 | -0.121 | -0.333, 0.091 | 0.263 |
|  | Digit Span | Patients | 131 | -0.278 | -1.25, 0.696 | 0.576 |
|  |  | Relatives | 53 | 1.45 | -0.350, 3.25 | 0.115 |
|  |  | Controls | 1086 | 0.0206 | -0.167, 0.208 | 0.830 |
|  | RAVLT Immediate Recall | Patients | 597 | 0.143 | -0.098, 0.384 | 0.243 |
|  |  | Relatives | 516 | -0.09 | -0.392, 0.212 | 0.561 |
|  |  | Controls | 793 | -0.117 | -0.291, 0.057 | 0.189 |
|  | RAVLT Delayed Recall | Patients | 595 | 0.055 | -0.0566, 0.167 | 0.333 |
|  |  | Relatives | 513 | 0.043 | -0.104, 0.190 | 0.565 |
|  |  | Controls | 781 | -0.063 | -0.146, 0.019 | 0.133 |
|  |  |  |  |  |  |  |
| Genes affected by deletions (deletion burden) | Block Design | Patients | 420 | -0.909 | -2.13, 0.309 | 0.143 |
|  |  | Relatives | 502 | -0.022 | -1.14, 1.09 | 0.969 |
|  |  | Controls | 1825 | -0.413 | -0.843, 0.017 | 0.060 |
|  | Digit Span | Patients | 131 | 0.383 | -1.533, 2.30 | 0.695 |
|  |  | Relatives | 53 | 0.736 | -1.77, 3.25 | 0.565 |
|  |  | Controls | 1086 | -0.195 | -0.666, 0.275 | 0.416 |
|  | RAVLT Immediate Recall | Patients | 597 | -0.092 | -0.528, 0.343 | 0.678 |
|  |  | Relatives | 516 | -0.333 | -0.971, 0.305 | 0.307 |
|  |  | Controls | 793 | -0.329 | -0.718, 0.060 | 0.098 |
|  | RAVLT Delayed Recall | Patients | 595 | -0.07 | -0.272, 0.132 | 0.498 |
|  |  | Relatives | 513 | -0.215 | -0.519, 0.089 | 0.165 |
|  |  | Controls | 781 | -0.160 | -0.345, 0.025 | 0.090 |
|  |  |  |  |  |  |  |
| Genes affected by duplications (duplication burden) | Block Design | Patients | 420 | 0.366 | -0.338, 1.07 | 0.308 |
|  |  | Relatives | 502 | -0.163 | -0.733, 0.408 | 0.576 |
|  |  | Controls | 1825 | -0.027 | -0.269, 0.215 | 0.826 |
|  | Digit Span | Patients | 131 | -0.465 | -1.55, 0.615 | 0.399 |
|  |  | Relatives | 53 | 1.28 | -1.02, 3.59 | 0.275 |
|  |  | Controls | 1086 | 0.060 | -0.142, 0.261 | 0.561 |
|  | RAVLT Immediate Recall | Patients | 597 | 0.246 | -0.042, 0.533 | 0.094 |
|  |  | Relatives | 516 | -0.019 | -0.359, 0.321 | 0.912 |
|  |  | Controls | 793 | -0.063 | -0.256, 0.131 | 0.526 |
|  | RAVLT Delayed Recall | Patients | 595 | 0.110 | -0.024, 0.243 | 0.107 |
|  |  | Relatives | 513 | 0.121 | -0.046, 0.287 | 0.155 |
|  |  | Controls | 781 | -0.039 | -0.131, 0.053 | 0.409 |
| Associations between known schizophrenia-associated CNVs and CNV burden with cognitive performance, only looking at one patient group at the time, either patients with psychosis, unaffected relatives or controls. For the schizophrenia associated CNV analysis digit span was not examined as fewer than 10 CNV carriers had available data. CNV burden was measured as number of genes affected by CNVs larger than >200 Kb, with <1% frequency. | | | | | | |
|  |  |  |  |  |  |  |
|  |  |  |  |  |  |  |

## Supplementary Table 11: Association between the CNV measures and clinical group

| **CNV Measure** | **Group** | **Schizophrenia-associated CNV carriers N (%)** | **Odds Ratio** | **95% CI** | **P-value** |
| --- | --- | --- | --- | --- | --- |
| Schizophrenia-associated CNVs | Control | 16 (0.8%) | 1 |  |  |
|  | Relative | 2 (0.3%) | 0.3 | -4.0, 4.5 | 0.086 |
|  | Patient | 12 (1.5%) | 1.3 | -1.8, 4.4 | 0.561 |
|  |  |  |  |  |  |
|  |  | **Number of observations** | **Odds Ratio** | **95% CI** | **P-value** |
|  |  |  |  |  |  |
| Genes affected (total CNV burden) | Patient vs Control | 2780 | 1.0 | -1.0, 3.0 | 0.516 |
|  | Relative vs Control | 2658 | 1.0 | -1.0, 3.0 | 0.307 |
|  |  |  |  |  |  |
| Genes affected (deletion burden) | Patient vs Control | 2780 | 1.0 | -1.0, 3.0 | 0.516 |
|  | Relative vs Control | 2658 | 1.0 | -1.0, 3.0 | 0.143 |
|  |  |  |  |  |  |
| Genes affected (duplication burden) | Patient vs Control | 2780 | 1.0 | -1.0, 3.0 | 0.784 |
|  | Relative vs Control | 2658 | 1.0 | -1.0, 3.0 | 0.672 |
| All analyses were adjusted for gender, age, centre and genetic relatedness (kinship matrix). CNV burden was measured as number of genes affected by CNVs larger than >200 Kb, with <1% frequency. | | | | | |

## **Supplementary Table 12:** Association between clinical group and cognitive performance

| Cognitive Measure |  | N | Mean (SD) | Regression Coefficient | 95% CI | P-value |
| --- | --- | --- | --- | --- | --- | --- |
| Block Design | Control | 1824 | 59.9 (21.3) | - | - | - |
|  | Relative | 502 | 51.7 (25.9) | -6.79 | -9.3, -4.2 | <0.0001 |
|  | Patient | 419 | 53.0 (27.9) | -12.0 | -14.6, -9.45 | <0.0001 |
|  |  |  |  |  |  |  |
|  |  |  |  |  |  |  |
| Digit Span | Control | 1086 | 51.5 (14.6) | - | - | - |
|  | Relative | 53 | 41.7 (13.9) | -10.30 | -14.8, -5..8 | <0.0001 |
|  | Patient | 131 | 48.2 (17.8) | -12.3 | -16.2, -8.4 | <0.0001 |
|  |  |  |  |  |  |  |
|  |  |  |  |  |  |  |
| RAVLT Immediate Recall | Control | 792 | 57.6 (13.8) | - | - | - |
|  | Relative | 516 | 56.1 (14.0) | -1.94 | -3.5, -0.40 | 0.014 |
|  | Patient | 596 | 49.3 (14.4) | -11.3 | -12.9, -9.7 | <0.0001 |
|  |  |  |  |  |  |  |
|  |  |  |  |  |  |  |
| RAVLT Delayed Recall | Control | 780 | 19.2 (6.4) | - | - | - |
|  | Relative | 513 | 19.1 (6.5) | 0.31 | -1.0, 0.4 | 0.408 |
|  | Patient | 594 | 14.4 (6.8) | -4.7 | -5.5, -3.9 | <0.0001 |
| The control group is the reference category. All analyses control for age, gender, centre and genetic relatedness (kinship matrix) | | | | | | |
|  |  |  |  |  |  |  |

# Supplementary Figures

## Supplementary Figure 1: Identifying common CNV loci


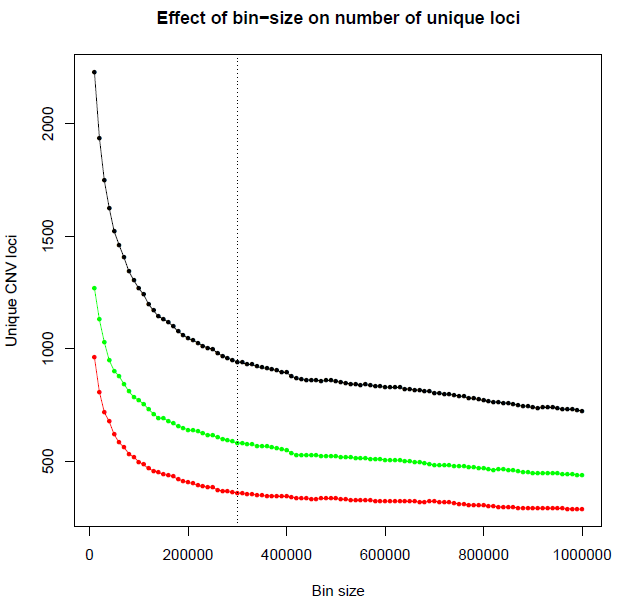


**Supplementary Figure 1:** Effect of bin-size on number of unique loci. Determining CNV frequency by independent mapping of start and stop positions falling within a determined stretch of DNA (bin size in base pairs). Effect of altering bin size (the maximum distance between individual CNVs’ start or stop positions for them to be considered as the same locus) on the number of unique independent CNV loci in the sample. Black = all CNVs, green = duplications only, red = deletions only. CNV algorithms estimate start and stop positions of CNVs in a non-exact way, and thus similar CNV loci may have slightly different start and stop positions in different carriers. This introduces a problem with determining exact CNV loci. In its extremes, either CNVs with slightly differing start and stop positions are collapsed into a single locus, or they are all treated as distinct loci. Here we tested the effect of altering the bin size used to map start and stop positions. As the bin size was increased, the number of unique independent CNV loci decreased. With smaller bin sizes, altering bin size had a large effect on the number of unique CNV loci, whereas with larger bin sizes, altering bin size had a smaller effect on the number of unique CNV loci. A bin size of 300 000 bp (indicated by the vertical dotted line) was chosen as a point where altering bin size began to have a smaller effect on the number on unique loci.

## Supplementary Figure 2: PRISMA Diagram

Records identified through database searching
(n = 996)

## Screening

## Included

## Eligibility

## Identification

Additional records identified through other sources
(n = 0)

Records after duplicates removed
(n = 996)

Records screened
(n = 996)

Records excluded
(n = 983)

Full-text articles assessed for eligibility
(n = 13)

Full-text articles excluded, with reasons
(n = 2)

Martin et al. 2014;224 excluded as it uses the same sample as Martin et al. 2014;272. Yeo et al. 2014 excluded as it uses the same sample as Yeo et al. 2013

Marti

Studies included in qualitative synthesis
(n = 11)

Studies included in quantitative synthesis (meta-analysis)
(n = 10)

Articles included in qualitative but not quantitative synthesis, with reasons

(n = 1)

Van Scheltinga et al. 2013, not included in quantitative synthesis as they did not report data in the form of correlations or standardised beta coefficients.

**Supplementary Figure 2:** PRISMA diagram displaying literature search results. Medline and PsychINFO were searched simultaneously using the Ovid platform. All of the relevant papers returned by the Ovid search of Medline and PsychINFO (905 results) were also returned by the PubMed search, therefore only the PubMed search is described here. Screening of titles and abstracts revealed 13 papers that were assessed for eligibility: 12 of these examined the association between CNV burden and intelligence^22,23,37,38,24–26,28–32^, and one ^27^ examined the difference in IQ between participants with and without large, rare CNVs. On request, Langley et al. 2011^27^ provided the results of correlations between CNV burden and IQ, allowing these results to be included in the meta-analysis, and Huguet et al. 2018^31^ provided standardised beta coefficients. Martin et al. 2014^37^ was not considered for inclusion, because the study uses the same sample of participants as Martin et al. 2014^26^, which performs a burden analysis. Yeo et al. 2014^38^ was not considered for inclusion as it used the same sample as Yeo et al. 2013^30^ This left ten papers which examined the association between CNV burden and IQ^22–30^.

## Supplementary Figure 3: Additional meta-analysis forest-plots

**
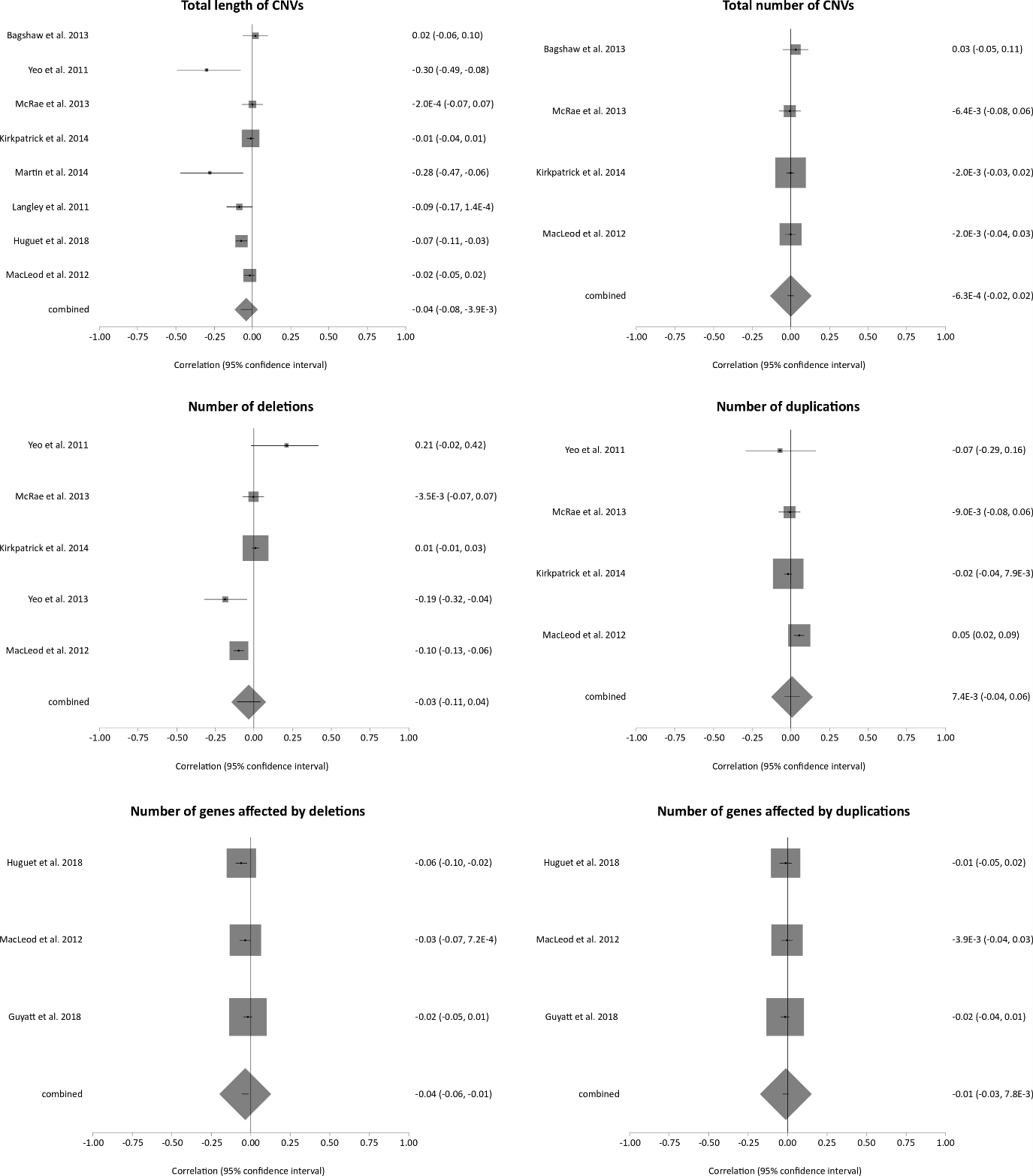
**

**Supplementary Figure 3:** Forest plots for the meta-analyses investigating length of all CNVs (N=10,132), number of all CNVs (N=10,699), number of deletions (N=10,395), number of duplications (N=10,206), genes affected by deletions (N=10,420) and genes affected by duplications (N=10,420).

# References

1 American Psychiatric Association. *Diagnostic and Statistical Manual of Mental Disorders - Fourth Edition. Text Revision*. 2006.

2 Andreasen NC, Flaum M, Arndt S. The Comprehensive Assessment of Symptoms and History (CASH). An instrument for assessing diagnosis and psychopathology. *Arch Gen Psychiatry* 1992; **49**: 615–23.

3 Spitzer RL, Williams JB, Gibbon M, First MB. The Structured Clinical Interview for DSM-III-R (SCID). I: History, rationale, and description. *Arch Gen Psychiatry* 1992; **49**: 624–9.

4 Endicott J, Spitzer RL. A diagnostic interview. The schedule for affective disorders and schizophrenia. *Arch Gen Psychiatry* 1978; **35: 837**–**84**: 837–844.

5 Wing JK, Babor T, Brugha T, Burke J, Cooper JE, Giel R *et al.* SCAN. Schedules for Clinical Assessment in Neuropsychiatry. *Arch Gen Psychiatry* 1990; **47: 589**–**59**: 589–593.

6 Wechsler D. *Wechsler Adult Intelligence Scale - Revised Manual.* 1981.

7 Wechsler D. *Wechsler Adult Intelligence Scale, Third Edition: Administration and Scoring manual.* 1997.

8 Rey A. *L’Examen clinique en psychologie*. 1964.

9 Crespo-Facorro B, Roiz-Santiáñez R, Pelayo-Terán JM, Rodríguez-Sánchez JM, Pérez-Iglesias R, González-Blanch C *et al.* Reduced thalamic volume in first-episode non-affective psychosis: correlations with clinical variables, symptomatology and cognitive functioning. *Neuroimage* 2007; **35**: 1613–23.

10 González-Blanch C, Crespo-Facorro B, Álvarez-Jiménez M, Rodríguez-Sánchez JM, Pelayo-Terán JM, Pérez-Iglesias R *et al.* Cognitive dimensions in first-episode schizophrenia spectrum disorders. *J Psychiatr Res* 2007; **41**: 968–977.

11 Johnstone EC, Ebmeier KP, Miller P, Owens DGC, Lawrie SM. Predicting schizophrenia: Findings from the Edinburgh high-risk study. *Br J Psychiatry* 2005; **186**: 18–25.

12 Korver N, Quee PJ, Boos HBM, Simons CJP, de Haan L, GROUP investigators. Genetic Risk and Outcome of Psychosis (GROUP), a multi-site longitudinal cohort study focused on gene-environment interaction: objectives, sample characteristics, recruitment and assessment methods. *Int J Methods Psychiatr Res* 2012; **21**: 205–21.

13 Walters JTR, Corvin A, Owen MJ, Williams H, Dragovic M, Quinn EM *et al.* Psychosis susceptibility gene ZNF804A and cognitive performance in schizophrenia. *Arch Gen Psychiatry* 2010; **67**: 692–700.

14 Waters F, Price G, Dragović M, Jablensky A. Electrophysiological brain activity and antisaccade performance in schizophrenia patients with first-rank (passivity) symptoms. *Psychiatry Res* 2009; **170**: 140–9.

15 Toulopoulou T, Goldberg TE, Mesa IR, Picchioni M, Rijsdijk F, Stahl D *et al.* Impaired intellect and memory: a missing link between genetic risk and schizophrenia? *Arch Gen Psychiatry* 2010; **67**: 905–13.

16 Bramon E, Pirinen M, Strange A, Lin K, Freeman C, Bellenguez C *et al.* A genome-wide association analysis of a broad psychosis phenotype identifies three loci for further investigation. *Biol Psychiatry* 2014; **75**: 386–397.

17 Wellcome T, Case T, Consortium C. Genome-wide association study of 14,000 cases of seven common diseases and 3,000 shared controls. *Nature* 2007; **447**: 661–678.

18 Marchini J, Howie B, Myers S, McVean G, Donnelly P. A new multipoint method for genome-wide association studies by imputation of genotypes. *Nat Genet* 2007; **39**: 906–913.

19 Wigginton JE, Abecasis GR. PEDSTATS: Descriptive statistics, graphics and quality assessment for gene mapping data. *Bioinformatics* 2005; **21**: 3445–3447.

20 Morris J a., Randall JC, Maller JB, Barrett JC. Evoker: A visualization tool for genotype intensity data. *Bioinformatics* 2010; **26**: 1786–1787.

21 Purcell S, Neale B, Todd-Brown K, Thomas L, Ferreira M a R, Bender D *et al.* PLINK: a tool set for whole-genome association and population-based linkage analyses. *Am J Hum Genet* 2007; **81**: 559–575.

22 Bagshaw ATM, Horwood LJ, Liu Y, Fergusson DM, Sullivan PF, Kennedy MA. No Effect of Genome-Wide Copy Number Variation on Measures of Intelligence in a New Zealand Birth Cohort. *PLoS One* 2013; **8**: 1–6.

23 Yeo RA, Gangestad SW, Liu J, Calhoun VD, Hutchison KE. Rare copy number deletions predict individual variation in intelligence. *PLoS One* 2011; **6**: 1–8.

24 McRae AF, Wright MJ, Hansell NK, Montgomery GW, Martin NG. No association between general cognitive ability and rare copy number variation. *Behav Genet* 2013; **43**: 202–207.

25 Kirkpatrick RM, McGue M, Iacono WG, Miller MB, Basu S, Pankratz N. Low-frequency copy-number variants and general cognitive ability: No evidence of association. *Intelligence* 2014; **42**: 98–106.

26 Martin AK, Robinson G, Reutens D, Mowry B. Copy number deletion burden is associated with cognitive, structural, and resting-state network differences in patients with schizophrenia. *Behav Brain Res* 2014; **272**: 324–334.

27 Langley K, Martin J, Agha SS, Davies C, Stergiakouli E, Holmans P *et al.* Clinical and cognitive characteristics of children with attention-deficit hyperactivity disorder, with and without copy number variants. *Br J Psychiatry* 2011; **199**: 398–403.

28 van Scheltinga AFT, Bakker SCC, van Haren NEM, Derks EMM, Buizer-Voskamp JEE, Cahn W *et al.* Schizophrenia genetic variants are not associated with intelligence. *Psychol Med* 2013; **43**: 2563–70.

29 MacLeod AK, Davies G, Payton A, Tenesa A, Harris SE, Liewald D *et al.* Genetic Copy Number Variation and General Cognitive Ability. *PLoS One* 2012; **7**. doi:10.1371/journal.pone.0037385.

30 Yeo RA, Gangestad SW, Liu J, Ehrlich S, Thoma RJ, Pommy J *et al.* The impact of copy number deletions on general cognitive ability and ventricle size in patients with schizophrenia and healthy control subjects. *Biol Psychiatry* 2013; **73**: 540–545.

31 Huguet G, Schramm C, Douard E, Jiang L, Labbe A, Tihy F *et al.* Measuring and Estimating the Effect Sizes of Copy Number Variants on General Intelligence in Community-Based Samples. *JAMA Psychiatry* 2018. doi:10.1001/jamapsychiatry.2018.0039.

32 Guyatt AL, Stergiakouli E, Martin J, Walters J, O’Donovan M, Owen M *et al.* Association of copy number variation across the genome with neuropsychiatric traits in the general population. Am. J. Med. Genet. Part B Neuropsychiatr. Genet. 2018. doi:10.1002/ajmg.b.32637.

33 Marshall CR, Howrigan DP, Merico D, Thiruvahindrapuram B, Wu W, Greer DS *et al.* Contribution of copy number variants to schizophrenia from a genome-wide study of 41,321 subjects. *Nat Genet* 2017; **49**: 27–35.

34 Stefansson H, Meyer-Lindenberg A, Steinberg S, Magnusdottir B, Morgen K, Arnarsdottir S *et al.* CNVs conferring risk of autism or schizophrenia affect cognition in controls. *Nature* 2014; **505**: 361–6.

35 Kirov G, Rees E, Walters JTR, Escott-Price V, Georgieva L, Richards AL *et al.* The penetrance of copy number variations for schizophrenia and developmental delay. *Biol Psychiatry* 2014; **75**: 378–85.

36 National Institute of Health. Quality Assessment Tool for Observational Cohort and Cross-Sectional Studies. https://www.nhlbi.nih.gov/health-pro/guidelines/in-develop/cardiovascular-risk-reduction/tools/cohort (accessed 18 Oct2016).

37 Martin AK, Robinson G, Reutens D, Mowry B. Cognitive and structural neuroimaging characteristics of schizophrenia patients with large, rare copy number deletions. *Psychiatry Res - Neuroimaging* 2014; **224**: 311–318.

38 Yeo RA, Gangestad SW, Walton E, Ehrlich S, Pommy J, Turner JA *et al.* Genetic influences on cognitive endophenotypes in schizophrenia. *Schizophr Res* 2014; **156**: 71–75.
